# Co-administration of iloprost and eptifibatide in septic shock (CO-ILEPSS)—a randomised, controlled, double-blind investigator-initiated trial investigating safety and efficacy

**DOI:** 10.1186/s13054-019-2573-8

**Published:** 2019-09-05

**Authors:** Rasmus Ehrenfried Berthelsen, Sisse Rye Ostrowski, Morten Heiberg Bestle, Per Ingemar Johansson

**Affiliations:** 1 Department of Anesthesiology and Intensive Care, Zealand University Hospital, Roskilde, University of Copenhagen, Copenhagen, Denmark; 2 Department of Clinical Immunology, Copenhagen University Hospital, Rigshospitalet, Blegdamsvej 9, 2100 Copenhagen Ø, Denmark; 3 Department of Clinical Medicine, University of Copenhagen, Blegdamsvej 3B, 2200 Copenhagen N, Denmark; 4 Section for Transfusion Medicine, Capital Region Blood Bank, Rigshospitalet, Blegdamsvej 9, 2100 Copenhagen Ø, Denmark; 5 Center for Systems Biology, University of Iceland, Sturlugata 8, 101 Reykjavik, Iceland; 6 Center for Translational Injury Research (CeTIR), Department of Surgery, University of Texas Health Science Center at Houston, 7000 Fannin, Suite 1800, Houston, TX 77030 USA

**Keywords:** Septic shock, Pathologic processes, Infection, Endothelium, Platelets, Iloprost, Eptifibatide, Platelet aggregation inhibitors

## Abstract

**Background:**

Part of the pathophysiology in septic shock is a progressive activation of the endothelium and platelets leading to widespread microvascular injury with capillary leakage, microthrombi and consumption coagulopathy. Modulating the inflammatory response of endothelium and thrombocytes might attenuate this vicious cycle and improve outcome.

**Method:**

The CO-ILEPSS trial was a randomised, placebo-controlled, double-blind, pilot trial. Patients admitted to the intensive care unit with septic shock were randomised and allocated in a 2:1 ratio to active treatment with dual therapy of iloprost 1 ng/kg/min and eptifibatide 0.5 μg/kg/min for 48 h or placebo. The primary outcomes were changes in biomarkers reflecting endothelial activation and disruption, platelet consumption and fibrinolysis. We compared groups with mixed models, post hoc Wilcoxon signed-rank test and Mann-Whitney *U* test.

**Results:**

We included 24 patients of which 18 (12 active, 6 placebo) completed the full 7-day trial period and were included in the per-protocol analyses of the primary outcomes. Direct comparison between groups showed no differences in the primary outcomes. Analyses of within-group delta values revealed that biomarkers of endothelial activation and disruption changed differently between groups with increasing levels of thrombomodulin (*p* = 0.03) and nucleosomes (*p* = 0.02) in the placebo group and decreasing levels of sE-Selectin (*p* = 0.007) and sVEGFR1 (*p* = 0.005) in the active treatment group. Platelet count decreased the first 48 h in the placebo group (*p* = 0.049) and increased from baseline to day 7 in the active treatment group (*p* = 0.023). Levels of fibrin monomers declined in the active treatment group within the first 48 h (*p* = 0.048) and onwards (*p* = 0.03). Furthermore, there was a significant reduction in SOFA score from 48 h (*p* = 0.024) and onwards in the active treatment group.

Intention-to-treat analyses of all included patients showed no differences in serious adverse events including bleeding, use of blood products or mortality.

**Conclusion:**

Our results could indicate benefit from the experimental treatment with reduced endothelial injury, reduced platelet consumption and ensuing reduction in fibrinolytic biomarkers along with improved SOFA score. The results of the CO-ILEPSS trial are exploratory and hypothesis generating and warrant further investigation in a large-scale trial.

**Trial registration:**

Clinicaltrials.com, NCT02204852 (July 30, 2014); EudraCT no. 2014-002440-41

**Electronic supplementary material:**

The online version of this article (10.1186/s13054-019-2573-8) contains supplementary material, which is available to authorized users.

## Introduction

Septic shock is a leading cause of death in the intensive care unit (ICU) with mortality rates above 40% [1, 2]. Treatment strategies consist of early recognition and diagnosis to facilitate timely initiation of antibiotic therapy and supportive care [3]. A series of pathogenic events are responsible for the transition from sepsis to septic shock. The initial reaction to infection is a neurohumoral, generalised pro- and anti-inflammatory response [4, 5] resulting in mobilisation and/or “spill over” of plasma substances and excessive cellular, coagulation and endothelial activation. The proinflammatory response induces widespread endothelial and microvascular injury resulting in disseminated intravascular coagulation with microvascular thrombosis, consumptive thrombocytopenia, coagulopathy, bleeding and a loss of endothelial integrity ultimately leading to capillary leakage, tissue oedema, tissue ischaemia and shock [5–7]. In the later stages of sepsis, immunodeficiency is a critical component of the pathology that causes multiple organ failure and death [8].

There are three major pathogenic pathways associated with the coagulopathy in sepsis: (1) tissue factor-mediated thrombin generation, (2) dysfunctional anticoagulant pathways and (3) blocked fibrinolysis [9]. Treatment strategies aimed at reducing coagulation activation with antithrombin [10], tissue factor pathway inhibitor [11] and activated Protein C [12, 13] have all failed to show improved survival in large clinical trials refuting this as a pathophysiological explanation.

The platelets and endothelium are interdependent in the vicious cycle of endothelial damage, microcirculatory failure, consumptive thrombocytopenia, coagulopathy, bleeding, immunodeficiency, tissue ischaemia, shock, organ failure and death, in patients with severe sepsis/septic shock. Selective targeting of either platelets or the endothelium may be sufficient to prevent the progressively more activated and damaged endothelium and activation of the platelets [14].

Prostacyclin is an endogenously produced molecule with anti-platelet, vasodilatory and cytoprotective properties released from the healthy endothelium as part of the natural anticoagulation system [15]. Intravenous prostacyclin in doses of 0.5–2.0 ng/kg/min has been reported to be successful at achieving endothelial modulating/preserving effects in patients with traumatic brain injury, without significant haemodynamic or platelet aggregation complications [16, 17].

Eptifibatide is a platelet glycoprotein (GP) IIb/IIIa receptor inhibitor that prohibits clot development in a predictable and easy controllable way. Inhibition of the GPIIb/IIIa receptor does not alter the paracrine function of platelets, which is considered a crucial part of maintaining vascular integrity and preventing haemorrhage in conditions with inflammation [18, 19]. Animal studies have reported that treatment with GPIIb/IIIa inhibitor protects against endothelial dysfunction in experimental endotoxemia [20, 21]. Furthermore, casuistic findings have shown that GPIIb/IIIa inhibition leads to clinically relevant thrombolysis in patients with mechanical prosthetic mitral valve thrombosis [22, 23].

The objective of the CO-ILEPSS trial was to investigate safety and efficacy of a combined infusion of low-dose prostacyclin (iloprost) and GPIIb/IIIa inhibitor (eptifibatide) for 48 h in patients with septic shock. We hypothesised that this dual treatment with iloprost and eptifibatide would deactivate the endothelium and restore vascular integrity, reduce formation of microvascular thrombosis and dissolve existing thrombi in the microcirculation and maintain platelet counts leading to improved platelet-mediated immune function and reduced risk of bleeding. Compared to the standard treatment (placebo), this was expected to translate into reduced organ failure and improved outcome in patients with septic shock.

## Methods

### Design

The CO-ILEPSS trial was an investigator-initiated single-centre randomised, placebo-controlled, double-blind phase 2a trial in patients with septic shock.

The trial was conducted from October 2014 to May 2016, and there were no significant changes to the trial protocol during the course of the trial. The trial is reported in accordance with the CONSORT statement [24], and a populated CONSORT checklist is available in Additional file 1. The trial was approved by the regional ethics committee, and all patients and/or their next of kin gave informed consent to participate. The full trial protocol is available in Additional file 2.

### Participants

Patients were allocated in a 2:1 ratio with 15 intention-to-treat (ITT) patients allocated to active treatment and 9 ITT patients allocated to control treatment (placebo). Patients who dropped out or were withdrawn from the trial prior to day 7 were replaced to ensure adequate data points, and 12 active treatment and 6 placebo patients, respectively, were treated as per protocol (PP). To replace patients, who were withdrawn, unblinded trial personal added envelopes containing the same allocation as the ones who dropped out and we recruited and re-randomised new patients.

We screened patients admitted to the intensive care unit (ICU) at Nordsjaellands Hospital (NOH) during the inclusion period. Patients were screened within 24 h of admission according to the following inclusion criteria:

- Adult intensive care patients (age ≥ 18 years)
- Sepsis, defined as suspected or confirmed site of infection or positive blood culture and ≥ 2 of 4 systemic inflammatory response syndrome (SIRS) criteria fulfilled within the last 24 h:

-  Temperature ≤ 36 °C or ≥ 38 °C
-  Heart rate ≥ 90 beats per minute
-  Mechanical ventilation for acute respiratory process or respiratory rate ≥ 20 breaths per minute or PaCO2 < 4.2 kPa
-  WBC ≥ 12,000/mm; OR ≤ 4000/mm OR > 10% bands

- Septic shock within the last 24 h, defined as:

-  Hypotension (MAP < 70 mmHg, lactate 4 mmol/l) despite ongoing resuscitation with fluids (crystalloids, colloids, blood products) within the last 24 h or
-  ≥ 30 ml/kg ideal body weight (IBW) fluid (crystalloids, colloids, blood products) given in the last 24 h and
-  Need for vasopressor/inotropic agents (noradrenaline, adrenaline or dopamine) within the last 24 h

- Can be randomised into trial and dosed < 24 h after septic shock diagnosis (the time point for the septic shock diagnosis corresponds to the time point where the vasopressor/inotropic therapy is initiated) and
- Consent is obtainable

A full description of the inclusion and exclusion criteria is provided in Additional file 3.

### Randomisation

The random allocation sequence was computer generated, and allocation pages were packed in sealed opaque envelopes. The envelopes were prepared by the principal investigator (SRO) at Rigshospitalet (RH) and delivered at the trial site NOH. At NOH, the envelopes were stored in a locked office at the post-anaesthesia care unit (PACU) located in a separate building from the ICU. The local investigators (REB and MHB) did not have access to this office. When a patient fulfilled inclusion criteria and consent had been obtained, randomisation was done by placing a phone call from the ICU to a nurse at the PACU. The nurse then opened the next envelope in line and prepared the trial drug or placebo according to the instructions. Syringes containing trial drug or placebo drug were then delivered to the investigator (REB) at the ICU where trial treatment was initiated.

### Intervention

Patients allocated to the active treatment arm received dual infusions of prostacyclin (iloprost) 1.0 ng/kg/min and GPIIb/IIIa inhibitor (eptifibatide) 0.5 μg/kg/min for 48 h. Iloprost and eptifibatide were both diluted in saline to a concentration with which the targeted treatment was achieved with an infusion of 4 ml/h. Treatment in the placebo group consisted of dual infusions of normal saline 4 ml/h for 48 h. The infusions of both active and placebo treatment were given either in two separate legs of a central venous catheter or in two separate peripheral venous catheters.

### Outcomes

The primary outcome of the CO-ILEPSS trial was divided in three different measures. These were:

1. Change in biomarkers indicative of endothelial activation and damage (sE-selectin, syndecan-1, soluble thrombomodulin (sTM), sVE-cadherin, nucleosomes, vascular endothelial growth factor (VEGF) and soluble vascular endothelial growth factor receptor 1 (sVEGFR1)) from baseline to 48 h post-randomisation
2. Change in platelet count from baseline to 48 h post-randomisation
3. Change in D-dimer and fibrin split products indicative of fibrinolysis (fibrin monomer complex, fibrin degradation products, D-dimer) from baseline to 48 h post-randomisation.

The reason for having three primary sub-endpoints was that they reflect different effects of active treatment on the vascular system that we wished to evaluate, i.e. endothelial activation, platelet consumption and fibrinolysis.

Secondary outcomes included severe bleeding (intracranial or clinical bleeding with the use of 3 RBC units or more/24 h); use of blood products in the ICU post-randomisation; mortality at days 7, 30 and 90; changes in Sequential Organ Failure Assessment (SOFA) score from baseline; and days of vasopressor, mechanical ventilation and renal replacement therapy (RRT) post-randomisation.

### Sample size/power calculation

The sample size for the CO-ILEPSS trial was not based upon a power calculation because there were no available data on the specific active dual drug therapy vs placebo in patients with septic shock.

However, in a previous study of safety and efficacy of prostacyclin vs placebo in patients undergoing Whipple surgery, post-operative levels of sVE-cadherin increased 1978 ± 461 pg/ml in the placebo group [25]. Based on this, we would be able to detect a difference of 33% in sVE-cadherin increase between groups with 12 patients in the active treatment group vs 6 patients in the placebo group, with a power of 0.8 and an alfa of 0.05.

### Blinding

The CO-ILEPSS trial was a double-blind, placebo-controlled trial, and all participants, next of kin, caregivers, investigators and sponsors were blinded for the trial allocation.

Both trial medications were colourless when diluted in saline, and it was impossible to distinguish the syringes with trial medicine from those with saline. Since the number of patients in the different groups was unequal, it was not possible to maintain blinding during the statistical analyses, but these were conducted according to the statistical analysis plan generated as part of the trial protocol.

### Biomarker analyses

Blood samples for the analyses of biomarkers were drawn pre-study drug/placebo administration, post 6 h, 24 h, 48 h, 72 h and 120 h. All samples were transferred to the local Blood Bank at the trial site for further processing (centrifugation, plasma aliquoting and freezing) and stored at − 80 °C for later analyses at the Dept. of Clinical Immunology, Blood Bank, Copenhagen University Hospital, Rigshospitalet. In brief, biomarkers were analysed by commercially available enzyme-linked immunosorbent assay (ELISA) according to the manufacturer’s recommendations: Syndecan-1 (Diaclone SAS, Besancon, France; lower limit of detection (LLD) 4.94 ng/ml), soluble thrombomodulin (sTM, Diaclone SAS, Besancon, France; LLD 0.31 ng/ml), soluble E-selectin (sE-selectin, sCD62E, Diaclone SAS, Besancon, France; LLD < 0.5 ng/ml), vascular endothelial (VE) cadherin (VE-cadherin, R&D Systems, Europe, Ltd., Abingdon, UK; LLD 0.113 ng/ml), vascular endothelial growth factor (VEGF, R&D Systems, Europe, Ltd., Abingdon, UK; LLD 9 pg/ml), histone-associated DNA fragments (nucleosomes, Cell Death Detection ELISA^PLUS^, Roche Diagnostics GmbH, Mannheim, Germany; LLD relative percentage with a maximal standard included), fibrin monomer complex (Biocompare, LLD 1.56 μg/ml); fibrin degradation product (Cloud-Clone Corp., Houston, USA; LLD 0.69 ng/ml), and D-dimer (Sekisui Diagnostics, LLC, Stanford, CT, USA; LLD 2–4 ng/ml).

### Statistical analysis

Summary statistics of continuous variables are presented as median with interquartile range (IQR). Summary statistics of frequency tables are presented as *n* (%). *p* values < 0.05 are considered significant.

The primary outcomes were analysed for efficacy in PP analyses. The difference between treatment groups for continuous data was evaluated with the analysis of variance (mixed model) and post hoc pairwise comparisons of means. Furthermore, delta values (numerical change in variables between time points) within and between groups were compared by paired (Wilcoxon signed-rank test) and non-paired (Mann-Whitney *U* test) non-parametric tests.

Biomarker measurements are presented as absolute values in Figs. 2 and 3 and as relative changes in percentage from baseline in Additional file 4.

Secondary outcomes were analysed on an ITT basis. The differences between treatment groups for categorical data were evaluated with McNemar’s test (change over time), frequency tables and chi-square statistics. The difference between treatment groups for continuous data was evaluated using the analysis of variance (mixed model) followed by post hoc pairwise comparisons of means. If the assumption of normality was not fulfilled, non-parametric test and Wilcoxon rank-sum test were used. Statistical analysis was performed using SAS 9.1.3 SP4 (SAS Institute Inc., Cary, NC, US).

## Results

**Fig. 1:**
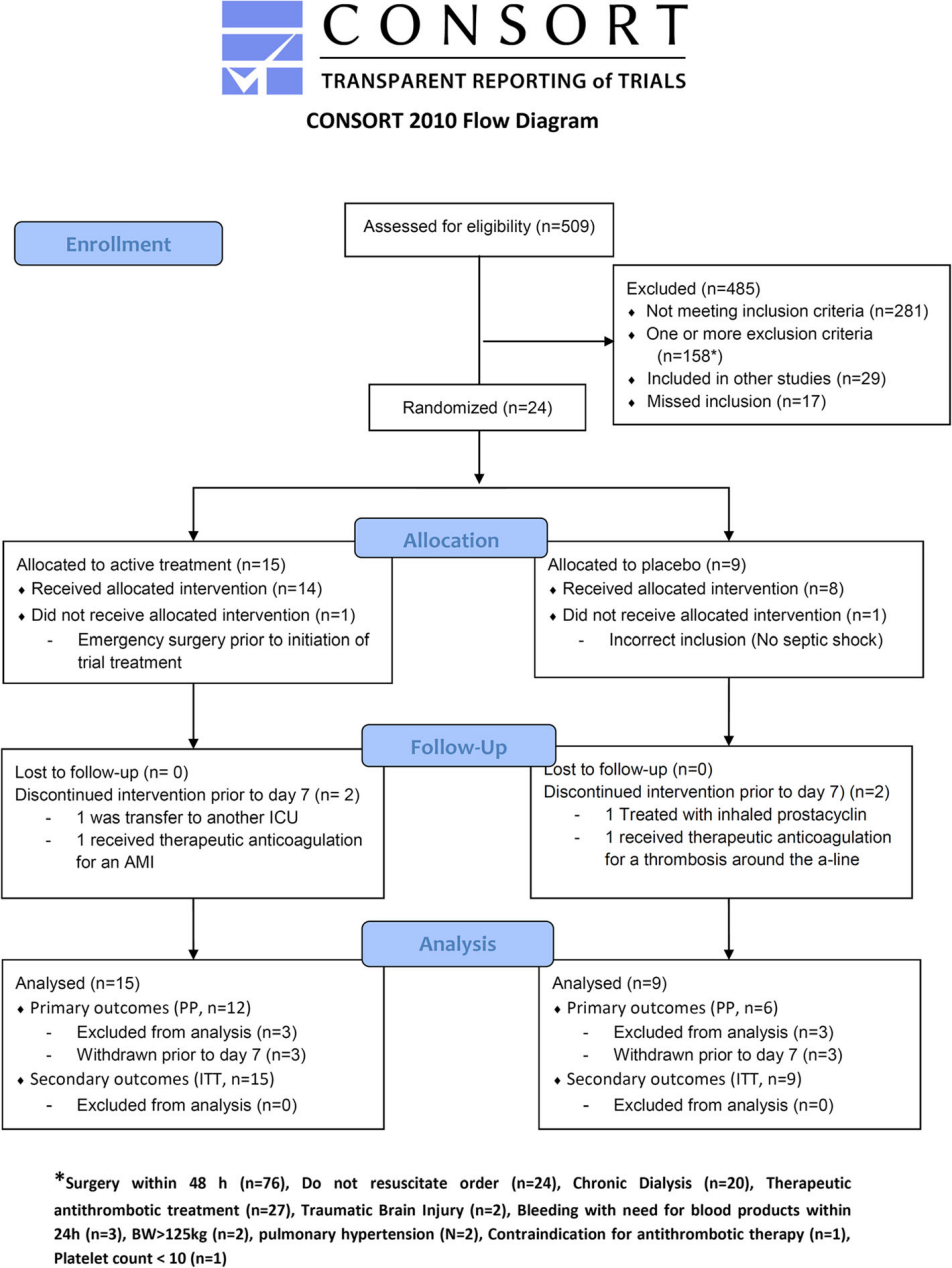
Consort flow diagram. ICU intensive care unit, AMI acute myocardial infarction, A-line arterial line, PP per protocol, ITT intention to treat

During the study period, we screened 509 patients and included 24. Most patients were excluded due to the absence of septic shock or completed/scheduled surgery within ± 48 h. Of the included patients, two patients were withdrawn prior to initiation of trial treatment, and four patients were withdrawn prior to day 7 (Fig. 1). These six patients were replaced in the trial. Reasons for withdrawal were incorrect inclusion (1), emergency surgery (1), transfer to another ICU (1), therapeutic anticoagulation therapy (2) and treatment with inhaled prostacyclin (1).

Table 1 shows baseline characteristics, use of organ supportive therapy and outcomes of patients included in the PP analyses. Only alkaline phosphatase was significantly different between groups at baseline, and it is worth noting that the disease severity was considerable with SOFA scores of 8–10, SAPS II scores of 46–48 and an observed 90-day mortality of 25–50%.

**Table 1:** Baseline characteristics, use of organ support and outcome of patients included in the per-protocol analyses

Variable	Active treatment	Placebo treatment
	Median (IQR)	Median (IQR)
*n*	12	6
Age, years	61.5 (54.2–68.6)	71.3 (64.2–75)
Male gender, *n* (%)	8 (66.7%)	6 (100%)
BMI, kg/m^2^	24.5 (22.1–28.7)	27.8 (22.8–30.4)
Infectious focus		
CNS, *n* (%)	0 (0%)	0 (0%)
Lungs, *n* (%)	10 (83.3%)	4 (66.7%)
Gastrointestinal, *n* (%)	0 (0%)	2 (33.3%)
Urogenital, *n* (%)	0 (0%)	0 (0%)
Skins and soft tissue, *n* (%)	2 (16.7%)	1 (16.7%)
Blood, *n* (%)	0 (0%)	1 (16.7%)
Unknown, *n* (%)	1 (8.3%)	0 (0%)
Identified pathogen (microbiology), *n* (%)	9 (75%)	5 (83.3%)
Comorbidity		
Chronic diagnosis, *n* (%)	9 (75%)	6 (100%)
Admitted from		
Acute medical care unit, *n* (%)	6 (50%)	3 (50%)
Medical department, *n* (%)	3 (25%)	1 (16.7%)
Disease severity, physiology and biochemistry		
SOFA (first)	9.0 (7.8–12.0)	9.5 (7.3–13.3)
SAPS II	46 (42–57)	48 (39–55)
Systolic blood pressure, mmHg	113 (100–120)	123 (104–145)
Lactate, mmol/l	1.4 (1.3–3.1)	3.5 (1.9–3.9)
pH	7.35 (7.3–7.41)	7.41 (7.39–7.41)
Haemoglobin, mmol/l	6.9 (6.4–7.7)	6.5 (5.8–7)
White blood cell count, × 10^9^/l	15 (9.8–19.1)	17.2 (8.8–23.1)
Platelet count, ×10^9^/l	188 (130–255)	212 (149–295)
INR	1.2 (1.1–1.2)	1.3 (1.1–2)
Antithrombin	0.64 (0.52–0.76)	0.58 (0.37–0.82)
D-dimer	4.6 (2.4–19.9)	14.4 (3.4–15.3)
Creatinine, μmol/l	97 (67–242)	126 (114–189)
Blood urea nitrogen (BUN), mmol/l	7.9 (5.3–13.2)	13.5 (8.6–17.9)
ALAT, U/l	35 (26–62)	35 (23–169)
Bilirubin, μmol/l	11 (5–24)	16 (8–29)
Basic phosphatase, U/l	64 (57–84)	149 (76–242)
C-reactive protein, mg/l	172 (129–244)	138 (89–169)
Therapy and outcome		
Ventilator days, *n* (%)	5 (3–6)	6 (4–6)
Ventilator-free days, *n* (%)	25 (24–27)	24 (6–24)
RRT days, *n* (%)	0 (0–0)	0 (0–0)
RRT-free days, *n* (%)	30 (29–30)	30 (8–30)
Vasopressor days, *n* (%)	5 (5–5)	5 (4–5)
Vasopressor-free days, *n* (%)	25 (24–25)	25 (6–25)
Discharged to admitting dept., *n* (%)	9 (75%)	3 (50%)
Discharged to other hosp., *n* (%)	1 (8.3%)	1 (16.7%)
Discharged to other ICU, *n* (%)	1 (8.3%)	1 (16.7%)
Active stop therapy, *n* (%)	2 (16.7%)	1 (16.7%)
7-day mortality, *n* (%)	0 (0%)	1 (16.7%)
30-day mortality, *n* (%)	1 (8.3%)	2 (33.3%)
90-day mortality, *n* (%)	3 (25%)	3 (50%)

*IQR* interquartile range, *BMI* body mass index, *CNS* central nervous system, *SOFA* Sequential Organ Failure Assessment, *SAPS II* Simplified Acute Physiology Score II, *INR* international normalised ratio, *ALAT* alanine aminotransferase, *RRT* renal replacement therapy, *ICU* intensive care unit, *NS* non-significant

### Primary outcomes

The PP primary analysis included data from the 18 patients (12 active and 6 placebo) who completed the full 7 days of the trial.

### Endothelial disruption biomarkers

**Fig. 2:**
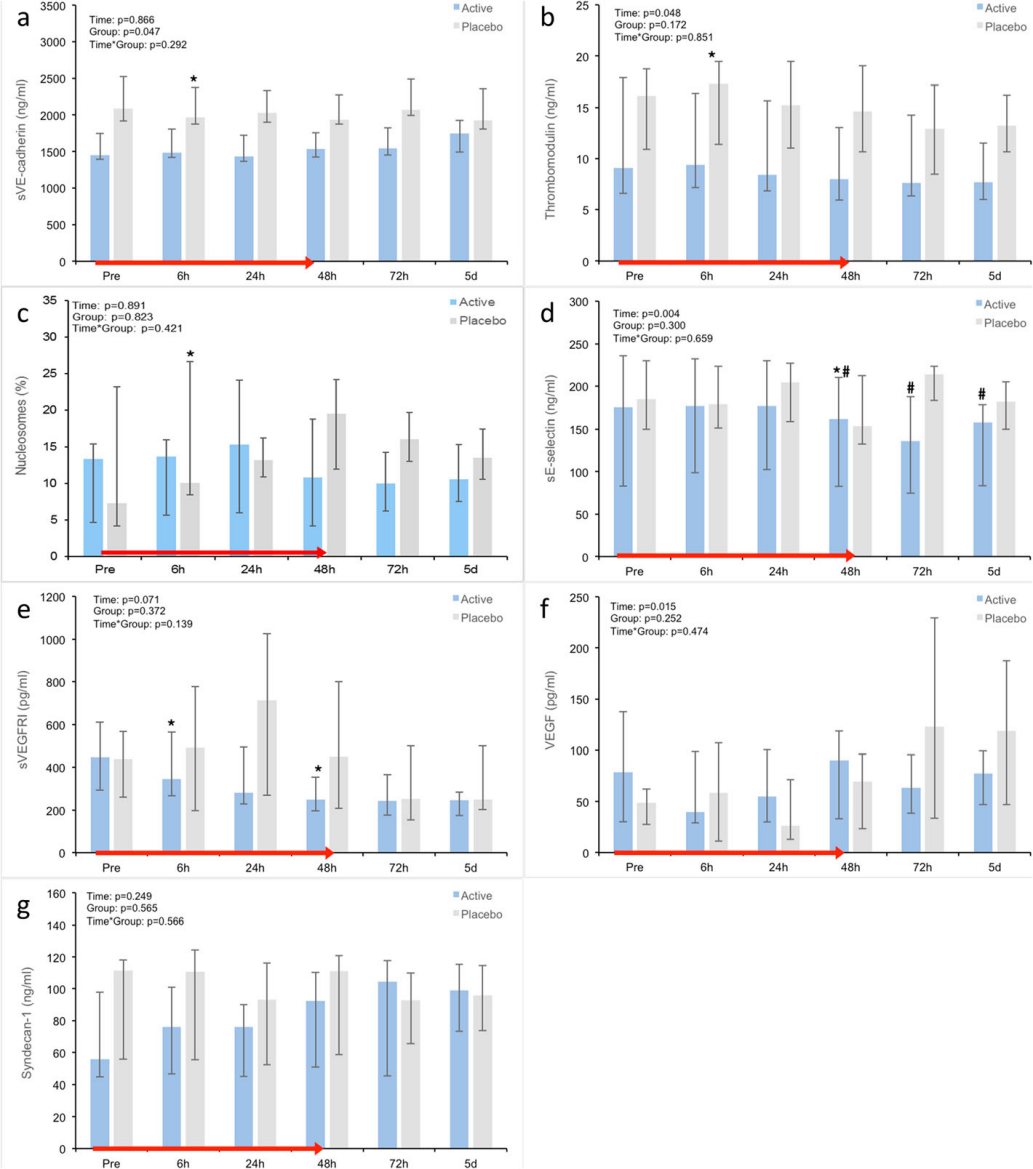
**a**–**g** Comparison of time-dependent changes in absolute biomarker values. Data shown as median (IQR). *Within-group change from baseline, *p* < 0.05; ^#^within-group change from non-baseline, *p* < 0.05. sVEGFRI soluble vascular endothelial growth factor receptor 1

At baseline, sVE-cadherin was significantly higher in the placebo group (*p* = 0.047) (Fig. 2a). Apart from this, there were no differences in the measured biomarkers between groups at baseline or at any time point during the 5-day follow-up (Fig. 2a–f). There were, however, differences in the within-group changes over time: At 6 h, there was a significant increase in both sTM (*p* = 0.03) and nucleosomes (*p* = 0.02) (Fig. 2b, c) only in the placebo group. Furthermore, in the placebo group, there was a tendency towards increasing levels of nucleosomes for up to 72 h (*p* = 0.06) (Fig. 2c).

At 48 h and throughout day 5, there was a significant decrease in sE-Selectin (*p* = 0.007) and sVEGFR1 (*p* = 0.005) only in the active treatment group (Fig. 2d, e).

### Platelet count

**Fig. 3:**
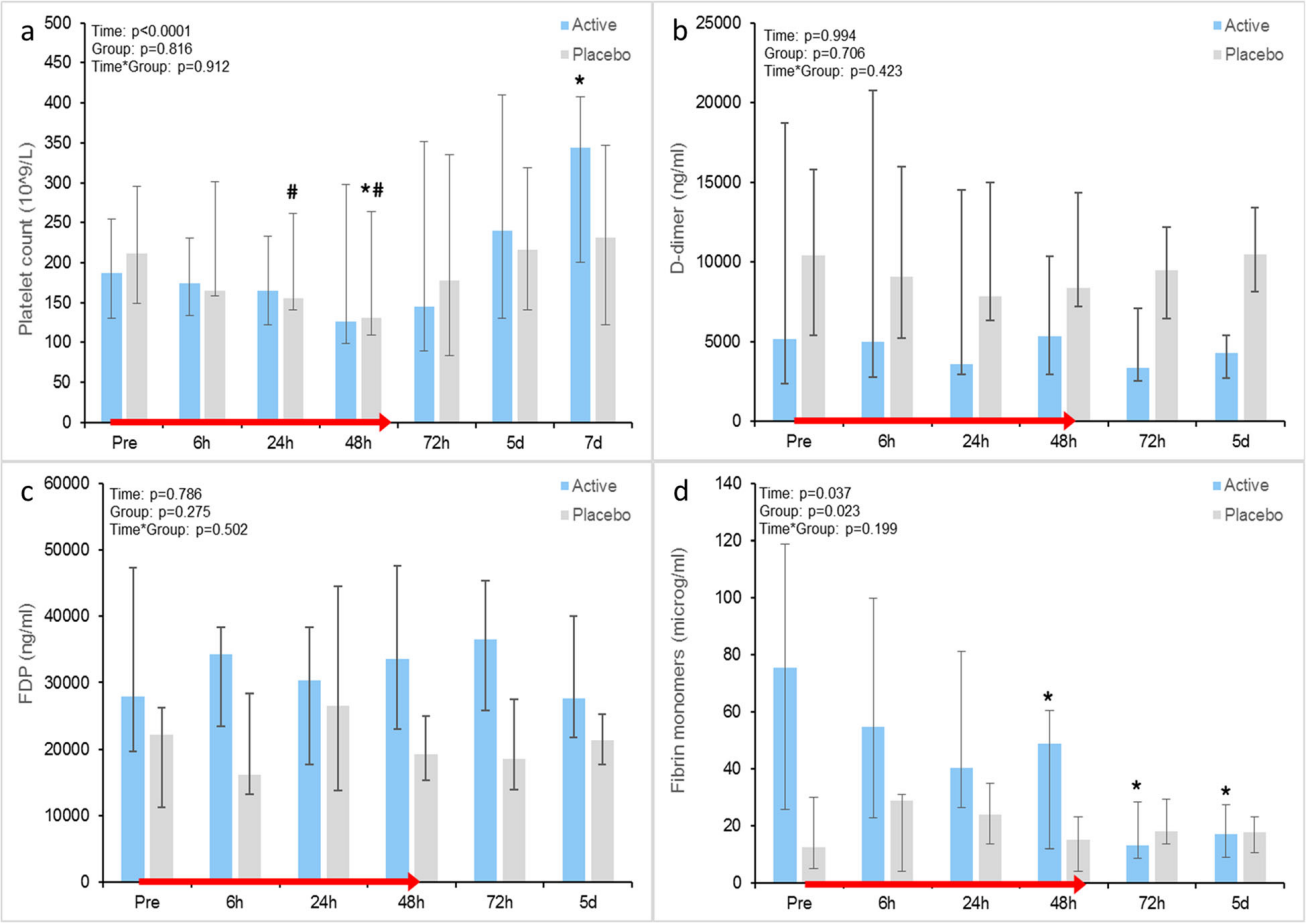
**a**–**d** Comparison of time-dependent changes in platelet count, D-dimer, Fibrin monomers and SOFA score. Data shown as median (IQR). *Within-group change from baseline, *p* < 0.05; ^#^within-group change from non-baseline, *p* < 0.05. SOFA Sequential Organ Failure Assessment

The platelet count did not differ significantly between groups at any time point during the trial. Similarly to the endothelial disruption biomarkers, there were differences in the within-group changes over time with a decline from baseline to 48 h only in the placebo group (*p* = 0.049) and an increase from baseline to day 7 only in the active treatment group (*p* = 0.023) (Fig. 3a).

### Fibrinolytic biomarkers

D-dimer and fibrinogen degradation products were similar in both groups (Fig. 3b, c). Levels of fibrin monomers were higher in the active treatment group than in the placebo group at baseline. Comparison of within-group delta values showed a significant decline within the first 48 h (*p* = 0.048) and onwards only in the active treatment group (time effect *p* = 0.04) (Fig. 3d).

### Secondary outcomes

In the ITT secondary analyses, a total of 24 patients (15 active and 9 placebo) were included.

All secondary endpoints including safety measures of bleeding, use of blood products in the ICU and mortality were comparable between groups (Additional file 5). Additionally, there was no difference in the occurrences of serious adverse events (SAEs) between groups and there were no suspected unexpected serious adverse reactions (SUSARs) in either group. Occurrences of SAEs and reasons for withdrawal and exclusions are summarised in Table 2.

**Table 2:** SAE/withdrawal/exclusion

ITT/PP	Allocation [duration]	SAE/withdrawal/exclusion	Outcome
ITT	Placebo [0 h]	Recovery with extubation before trial drug administration (incl. criteria not met). Excluded	Death on day 13 (resp. failure)
ITT	Placebo [14 h]	Thrombosis in the arterial cannula (difficult insertion in Aa. radialis, instead US-guided insertion in a. brachialis). SAE/withdrawal	LMWH treatment (10.000 IE) with good effect. Survivor
ITT	Placebo [14 h]	Indication for Flolan inhalation (severe respiratory failure). SAE/withdrawal	Death on day 2 (respiratory failure)
PP	Placebo [completed]	Iatrogenic pneumothorax and intraabdominal bleeding after liver abscess drainage, 28 h after ceasing trial drug. SAE	Death on day 2 (pneumonia)
ITT	Active [0 h]	Indication for acute abdominal surgery before trial drug administration. Excluded	Survivor
ITT	Active [21 h]	Indication for therapeutic LMWH (suspicion of type II MI). Cardiac enzymes elevated *before* inclusion, but increasing. SAE/withdrawal	Death on day 10 (cardiac failure)
ITT	Active [28 h]	Transferred to other ICU due to overcrowding. Withdrawal	Survivor
PP	Active [complete]	Severe septic shock (*Capnocytophagus carnimorsus*) with bradycardia treated with 1 mg adrenalin IV and 2 min CPR. Circulatory stabilised after 2 min. Transferred to tertiary ICU day 4. Necrotomy of the face and leg day 27. Finger amputation (1., 2., 4., 5. finger on the left hand) day 35. Femur amputation day 50. SAE	Survivor

*ITT* intention to treat, *PP* per protocol, *SAE* serious adverse event, *US* ultrasound, *LMWH* low molecular weight heparin, *MI* myocardial infarction, *ICU* intensive care unit, *IV* intravenous, *CPR* cardiopulmonary resuscitation

Comparison of within-group changes over time revealed a significant reduction in SOFA score at 48 h (*p* = 0.024) and onwards in the active treatment group, but not in the placebo group.

## Discussion

### Main findings

Dual therapy with iloprost and eptifibatide for 48 h in patients with septic shock had no significant effect on absolute values of biomarker levels compared to placebo treatment.

Analysis of within-group changes over time revealed biomarker changes indicative of reduced endothelial injury, reduced fibrinolysis and reduced platelet consumption in the active treatment group. This was accompanied by an overall decline in SOFA score from 48 h and onwards that was not observed in the placebo-treated patients. Additionally, the trial treatment was not associated with increased bleeding or the use of blood products as compared to placebo.

### Endothelial injury

Taken together, the observed changes in sTM, nucleosomes, sE-selectin, VEGF and sVEGFR1 could reflect reduced levels of endothelial activation, disruption and cell damage.

Prostacyclin doses, corresponding to the low dose chosen for this trial (1.0 ng/kg/min), have previously been demonstrated not to increase bleeding risk or haematoma size in patients with traumatic brain injury [17] and to reduce the need for blood transfusion in patients undergoing Whipple surgery due to pancreatic cancer [25]. Our results are in alignment with these former trials which also demonstrated beneficial effects of iloprost on vascular integrity in critically ill patients reflected by similar changes in sE-selectin, sTM and nucleosomes [17, 25, 26].

Endothelial protection could be ascribed to the cytoprotective effects of prostacyclin, which in its endogenous form induces a reduction in inflammation and stabilisation of lysosomal and cell membranes [27]. The dose of 1.0 ng/kg/min is approximately five- to tenfold higher than the normal endogenous production of prostacyclin from the healthy endothelium [28], and we expected this to restore vascular integrity in septic patients with endothelial injury and dysfunction.

The clinical impact of within-group changes in these biomarkers remains to be seen, but observational data have linked increased levels of sTM to reduced survival in patients with septic shock [29].

### Platelet protection and thrombotic activity

The increasing platelet count in the active treatment group could indicate protective effect against platelet consumption. In a previous study by Link et al., it was demonstrated that administration of a platelet GPIIb/IIIa receptor inhibitor in combination with unfractionated heparin for 96 h was tolerated in patients with cardiogenic shock and need for dialysis. Importantly, in this study, treatment with the GPIIb/IIIa receptor inhibitor was not associated with increased bleeding but was rather associated with a significantly lower number of platelet transfusions and a higher, maintained platelet count, as compared to controls anticoagulated with heparin alone [30]. This finding of preserved platelet count is in accordance with the finding in our study.

In addition to preserved platelet counts, the inhibition of platelet-monocyte aggregation might have an anti-inflammatory effect, and thus serve to protect the endothelium and microvasculature [31]. A reduced thrombotic activity was reflected in declining levels of fibrin monomers in the active treatment group.

### Safety

The individual doses of iloprost and eptifibatide selected for this trial are lower than the recommended doses for their respective approved indications. The dosages chosen for the current trial are in alignment with doses that have been reported to result in the desired effect for each agent without causing significant adverse side effects [17, 25, 30].

The safety of the co-administration of eptifibatide and iloprost in a dose comparable to the dose applied in the present study (eptifibatide 0.5 μg/kg/min + iloprost 1.0 ng/kg/min infused for 24 h) is supported by a completed phase I/II trial in patients undergoing primary PCI due to ST-elevated myocardial infarct [26]. This trial found no bleeding-related adverse events, and no treatment-related adverse events occurred.

### Limitations

Our main inclusion criterion was septic shock defined as the use of norepinephrine in patients with sepsis. This ensured that the screening and inclusion process was rather pragmatic and easy to perform, but we might have limited the potential effect of our intervention, since it is not given that all patients with septic shock have equal degrees of endothelial dysfunction and/or consumption coagulopathy. If we had used one or more specific markers in our screening, we might have been able to show higher efficacy of our trial treatment.

The CO-ILEPSS pilot trial is exploratory and hypothesis generating. Our small sample size and the single-centre design limit both external validity and the ability to draw any definitive conclusions from our results. The lack of a power calculation might have limited our ability to detect a difference in our primary outcome. Furthermore, our primary outcome is a composite of three categories of biomarkers with a total of 11 sub-components, which poses a problem of multiplicity.

## Conclusion

The results of the CO-ILEPSS trial suggest that dual therapy with iloprost and eptifibatide for 48 h in patients with septic shock is safe and may even be beneficial. Biomarker measurements indicated reduced endothelial activation, disruption and cell damage, accompanied by less severe thrombocytopenia. Interestingly, the dual treatment resulted in significant reductions in SOFA score after 48 h compared to placebo treatment. Future adequately powered trials are warranted to reveal if this treatment may lead to improved patient-centred outcomes.

## Additional files

Additional file 1: Populated CONSORT checklist. (PDF 65 kb)

Additional file 2: Trial protocol. (PDF 662 kb)

Additional file 3: In- and exclusion criteria. (PDF 369 kb)

Additional file 4: Relative biomarker measures. (PDF 540 kb)

Additional file 5: Secondary endpoints. (PDF 640 kb)

## Data Availability

The datasets used and analysed during the current study are available from the corresponding author on reasonable request.

## Abbreviations

CO-ILEPSS: Co-administration of iloprost and eptifibatide in septic shock
ELISA: Enzyme-linked immunosorbent assay
GP: Glycoprotein
ICU: Intensive care unit
ITT: Intention to treat
IQR: Interquartile range
LLD: Lower limit of detection
MHB: Morten Heiberg Bestle
NOH: Nordsjaellands Hospital
SOFA: Sequential Organ Failure Assessment
PACU: Post-anaesthesia care unit
PIJ: Pär Ivar Johansson
PP: Per protocol
REB: Rasmus Ehrenfried Berthelsen
RRT: Renal replacement therapy
SAE: Serious adverse event
SAPS II: Simplified Acute Physiology Score
SAR: Serious adverse reaction
SRO: Sisse Rye Ostrowski
sTM: Soluble thrombomodulin
SUSAR: Suspected unsuspected serious adverse reaction
sVEGFR1: Soluble vascular endothelial growth factor receptor 1
VEGF: Vascular endothelial growth factor
