# Supplementary material for: Co-administration of iloprost and eptifibatide in septic shock (CO-ILEPSS)—a randomised, controlled, double-blind investigator-initiated trial investigating safety and efficacy
Source: Crit Care. 2019 Sep 5;23:301. doi: 10.1186/s13054-019-2573-8 (PMC6727583; doi:10.1186/s13054-019-2573-8)
Supplement: Supplementary file 2 — Trial protocol. (PDF 662 kb) [file 13054_2019_2573_MOESM2_ESM.pdf]

CO-ILEPSS trial

CO-administration of ILoprost and EPtifibatide in Septic Shock patients

EudraCT no. 2014-002440-41

"Safety and efficacy of iloprost and eptifibatide co-administration compared to standard therapy in patients with septic shock – a randomized, controlled, double-blind investigator-initiated trial"

**Steering committee**	Pär I. Johansson MD, DMSc, MPA<br>Section for Transfusion Medicine<br>Capital Region Blood Bank<br>Rigshospitalet, 2034<br>Blegdamsvej 9, DK-2100<br>Copenhagen, Denmark<br>per.johansson@regionh.dk
	Sisse R. Ostrowski, MD, PhD, DMSc<br>Section for Transfusion Medicine<br>Capital Region Blood Bank<br>Rigshospitalet, 2034<br>Blegdamsvej 9, DK-2100<br>Copenhagen, Denmark<br>sisse.ostrowski@gmail.com
	Morten Bestle MD, PhD<br>Department of Anesthesia and Intensive Care<br>Nordsjællands hospital<br>Dyrehavevej 29, DK-3400<br>Hillerød, Denmark<br>morten.bestle@regionh.dk
**Trial monitoring**	GCP-Unit - Copenhagen University Hospital<br>Bispebjerg Hospital, Building 51, 3.sal<br>Bispebjerg Bakke 23<br>DK-2400 Copenhagen, Denmark<br>Tel. +45 3531 3890
**Principal investigator**	Morten Bestle MD, PhD<br>Department of Anesthesia and Intensive Care<br>Nordsjællands hospital<br>Dyrehavevej 29, DK-3400<br>Hillerød, Denmark<br>Tel. +45 4829 7244<br>Mobile +45 4195 1195<br>morten.bestle@regionh.dk
**Trial site**	Department of Anesthesia and Intensive Care<br>Nordsjællands hospital<br>Dyrehavevej 29, DK-3400<br>Hillerød, Denmark<br>Tel. +45 4829 4213
**Sponsor**	Sisse R. Ostrowski, MD, PhD, DMSc<br>Section for Transfusion Medicine<br>Capital Region Blood Bank<br>Rigshospitalet, 2034<br>Blegdamsvej 9, DK-2100<br>Copenhagen, Denmark<br>sisse.ostrowski@gmail.com

Signature page

CO-ILEPSS trial

CO-administration of ILoprost and EPtifibatide in Septic Shock patients

EudraCT no. 2014-002440-41

"Safety and efficacy of iloprost and eptifibatide co-administration compared to standard therapy in patients with septic shock – a randomized, controlled, double-blind investigator-initiated trial"

Principal investigator
Morten Bestle MD, PhD
Department of Anesthesia and Intensive Care
Nordsjællands hospital

____
(Date and signature)

Sponsor
Sisse R. Ostrowski, MD, PhD, DMSc
Section for Transfusion Medicine
Capital Region Blood Bank
Rigshospitalet

____
(Date and signature)

Table of contents

CO-ILEPSS trial 1
Signature page 3
Table of contents 4
1. Medical emergency contact details 6
2. List of abbreviations 7
3. Introduction 8
3.1 Background 8
3.2 Rationale for the trial 8
3.3 Design rationale 9
3.3.1 Iloprost dose rationale – safety and efficacy 9
3.3.2 Eptifibatide dose rationale – safety and efficacy 10
3.3.3 Use of placebo rationale 10
3.4 Investigational products 10
3.3.1 Ilomedin® iloprost, prostacyclin analogue 10
3.3.2 Integrilin® eptifibatide, platelet GPIIb/IIIa receptor inhibitor 10
4. Trial objectives 10
4.1 Objectives 10
4.2 Hypothesis 11
5. Trial design 11
5.1 Trial flow diagram 11
5.2 Trial table of observations and blood sampling 12
5.3 Endpoints 12
5.1.1 Primary endpoints 12
5.1.2 Secondary endpoints 12
6. Patient selection and withdrawal 12
6.1 Inclusion criteria 12
6.2 Exclusion criteria 13
6.3 Removal of patients from the trial, therapy or assessment 13
6.4 Patient withdrawal 14
6.5 Trial discontinuation 14
7. Trial treatment 14
7.1 Identity of the investigational product 14
7.1.1 Packaging and labeling of the investigational product 14
7.1.2 Storage, issue, and return of investigational product 14
7.2 Description of the investigational product 15
7.2.1 Iloprost 15
7.2.2 Eptifibatide 15
7.3 Randomization 15
7.4 Preparation of trial study drug (active, placebo) 15
7.4.1 Iloprost (study drug 1) 15
7.4.2 Eptifibatide (study drug 2) 16
7.4.3 Placebo (study drug 1) 16
7.4.4 Placebo (study drug 2) 16
7.5 Dosage and administration of study drug 16
7.6 Medications causing withdrawal of patients during trial 17
7.7 Treatment compliance 17
8. Trial schedule of events 17
8.1 Patient eligibility 17
8.2 Patient information/Informed consent procedure 17
8.3 Treatment, observation and follow up 17
8.4 Extended follow-up 18
8.5 Recruitment period 18
8.6 Number of patients 18
8.7 Treatment allocation 18

8.8 Stopping rules 18
9. Trial assessments 18
9.1 Clinical assessments 18
9.1.1 Demographic data, medical history, and physical examination 18
9.1.2 Vital signs and weight 18
9.2 Laboratory assessments 18
9.2.1 Biochemistry and hematology 19
9.2.2 Endothelial and fibrinolysis biomarkers 19
9.2.4 Research biobank 19
9.3 The CRF 19
10. Serious adverse reactions 19
10.1 Assessment and registration of SARs/SAEs and SUSARs 20
10.2 Reporting of SARs/SAEs and SUSARs 20
11. Analysis of trial data 21
11.1 Endpoints 21
11.1.1 Primary endpoints 21
11.1.2 Secondary endpoints 21
11.1.3 Other endpoints 21
11.2 Definitions of evaluability 21
11.3 Statistical methods 21
11.3.1 Primary endpoints 22
11.3.2 Secondary endpoints 22
11.3.3 Other endpoints 22
11.4 Sample size and power 22
11.5 Source data and patient files 22
12. Ethical considerations 23
12.1 Independent ethical committee 23
12.2 Patient information and informed consent 23
12.3 Ethical and risk/benefit considerations 23
13. Monitoring and quality assurance (QA) 24
13.1 Compliance with Good Clinical Practice (GCP), national legislation and quality standards and local SOPs 24
13.2 Monitoring 24
13.3 Source data verification 24
13.4 CRF handling 25
13.5 Changes to the final protocol 25
13.6 Deviations from the trial protocol 25
14. Finances 25
15. Insurance 25
16. Publication of trial results 25
17. Trial organization 26
18. References 27

Appendix 1 30
Ilomedin SPC

Appendix 2 40
Eptifibatide SPC

1. Medical emergency contact details

Principal investigator	Morten Bestle<br>Mobile: +45 4195 1195
Life threatening bleeding emergencies	Pär I. Johansson<br>Mobile: +45 2372 9202
Notification of SUSARs	Sisse R. Ostrowski<br>Mobile: +45 2443 0464

2. List of abbreviations

AE	Adverse event
AR	Adverse reaction
CRF	Case report form
CRRT	Continuous renal replacement therapy
Endothelium	Single cell layer covering the luminal (inner) surface of all vessels in the body
GCP	Good clinical practice
GMP	Good manufacturing practice
GPIIb/IIIa rec. inh.	Anti-platelet drug that inhibits glycoprotein IIb/IIIa (the fibrin/fibrinogen receptor)
ICU	Intensive care unit
i.v	Intravenous
Iloprost	Prostacyclin analogue
LMWH	Low molecular weight heparin
PCI	Percutaneous coronary intervention
Prostacyclin	Endogenously produced eicosanoid (part of the natural anticoagulation system)
SAE	Serious adverse event
SAPS	Simplified Acute Physiology Score
SAR	Serious adverse reaction
Sepsis	Presence (probable or documented) of infection together with systemic manifestations of infection
Severe sepsis	Sepsis plus sepsis-induced organ dysfunction or tissue hypoperfusion
Septic shock	Severe sepsis with hypotension not reversed with fluid resuscitation and need for vasopressor/inotropic therapy
SOFA score	Sequential Organ Failure Assessment score
SPC	Summary of product characteristics
SUSAR	Suspected unexpected serious adverse reaction
Thrombocytopenia	Platelet count below normal
UFH	Unfractionated heparin

3. Introduction

3.1 Background

Patients with severe sepsis remain an important clinical challenge and an economic burden in intensive care. An estimated 750,000 cases of severe sepsis occur each year in the US and approximately 700,000 cases occur each year in the EU [Guidet et al. 2005; Angus and van der Poll 2013]. Recently it was reported that severe sepsis was identified in 35% of patients during their ICU admission [Irish Critical Care Group 2008] and the proportion of patients diagnosed with severe sepsis or septic shock increased from 2000 to 2012 [Iwashyna and Angus 2014]. The absolute number of deaths due to sepsis increased two-fold from 22 in 1979 to 44 deaths per 100,000 in 2000 and the incidence of septic patients with any organ failure also increased from 19% between 1979 and 1989 to 30% in 2000, pointing to a greater severity of illness among these patients. For patients with septic shock [Annane et al. 2003], data were extracted from the College des Utilisateurs de Bases de donnees en Reanimation (CUB-Rea) Network in France, including more than 100,000 ICU admissions in 22 hospitals over an 8-year period from 1993 to 2000. The results showed an increase in the incidence of septic shock, from 7 per 100 admissions in 1993 to 10 per 100 admissions in 2000, and an overall mortality of 56%.

Severe sepsis is defined as sepsis with dysfunction of one or more organs, incl. mental status, lungs, coagulation, renal, liver, or circulatory failure evidenced by hypoperfusion with lactic acidosis and shock. Septic shock is per definition sepsis with hypotension and/or lactate acidosis refractory to bolus fluids.

A series of pathogenic events are responsible for the transition from sepsis to severe sepsis/septic shock. The initial reaction to infection is a neurohumoral, generalized pro- and anti-inflammatory response [Nguyen et al. 2006; Angus and van der Poll 2013] resulting in mobilization and/or "spill over" of plasma substances and excessive cellular, coagulation and endothelial activation. The plasma substances include cytokines, interleukins, proteases, reactive oxygen and nitrogen species and platelet activating factor that, together with complement [Haeney et al. 2003] and coagulation cascade activation, amplify the proinflammatory response hereby inducing widespread endothelial and microvascular injury resulting in microvascular thrombosis, consumptive thrombocytopenia and coagulopathy, bleeding and a loss of endothelial integrity ultimately leading to in capillary leakage, tissue edema, tissue ischemia and shock [Aird WC 2003; Karimova et al. 2001; Angus and van der Poll 2013]. In the later stages of sepsis, immunodeficiency is a critical component of the pathology that causes multiple organ failure and death [Boomer et al. 2011].
There are three major pathogenetic pathways associated with the coagulopathy in sepsis: (1) tissue factor (TF)-mediated thrombin generation, (2) dysfunctional anticoagulant pathways, and (3) blocked fibrinolysis [Esmon 2002]. Treatment strategies aimed at reducing coagulation activation with antithrombin [Afshari et al. 2008] and tissue factor pathway inhibitor [Abraham et al. 2003] and activated Protein C [Abraham et al. 2005; Bernard et al. 2001] have all failed to show improved survival in large clinical trials.

3.2 Rationale for the trial

Severe sepsis and septic shock is associated with considerable morbidity and mortality and its incidence is steadily increasing but to date, no treatments other than life-support and antibiotics exist.
The rationale for the suggested combination therapy in this study with low-dose iloprost (targeting the endothelium) and eptifibatide (targeting the platelets), is that the platelets and the endothelium are interdependent in the vicious cycle of endothelial damage, microcirculatory failure, consumptive thrombocytopenia, coagulopathy, bleeding, immunodeficiency, tissue ischemia, shock, organ failure and death, in patients with severe sepsis/septic shock. Selective targeting of platelets or selective targeting of endothelial activation [Massberg et al. 2003] may be sufficient to prevent the progressively more activated state of both endothelium and platelets [Chen et al. 2013]. Furthermore, in a recent randomized study of patients suffering from frost bites (a condition with microvascular damage very similar to that observed in severe sepsis/septic shock), combination therapy with prostacyclin and a platelet inhibitor was the only intervention that best protected tissues and completely avoided amputations (0% vs. 40% digits amputated, $p<0.001$) [Cauchy et al. 2011].
The combination therapy is thus expected to deactivate the endothelium and restore vascular integrity (iloprost), reduce formation of microvascular thrombosis and dissolve existing clots in the microcirculation (eptifibatide, iloprost), maintain platelet counts (eptifibatide, iloprost) [Link et al. 2008; Windeløv et al. 2010] and thereby improving platelet-mediated immune function [Semple et al. 2011] and reducing risk of

bleeding [Windeløv et al. 2010]. This may translate into reduced organ failure and improved outcome in patients with severe septic shock.

Prostacyclin/iloprost is an eicosanoid i.e., an endogenously produced molecule with anti-platelet, vasodilatory and cytoprotective properties released from the healthy endothelium as part of the natural anticoagulation system [Davies and Hagen 1993]. It is used to treat pulmonary hypertension and severe atherosclerotic limp ischemia in doses typically ranging from 4 to >10 ng/kg/min. It has been reported to be the main endogenous platelet inhibitor as evaluated by platelet aggregation [Scheeren et al. 1997], but the effect of prostacyclin on clot formation in whole blood reveals no platelet inhibitory effects in doses up to 4 ng/kg/min in healthy volunteers [Johansson and Ostrowski, unpublished data]. This is in alignment with the fact that prostacyclin infusion (up to 4 ng/kg/min) is commenced perioperatively in the re-perfusion phase of liver transplantation without compromising hemostasis [Kishida et al. 1997]. Furthermore, doses corresponding to the low-dose chosen for this trial (1.0 ng/kg/min) do not increase bleeding risk or hematoma size in patients with traumatic brain injury [Naredi et al. 2001] and reduces need for blood transfusion in patients undergoing Whipple surgery due to pancreatic cancer [Johansson and Ostrowski, unpublished data].

Eptifibatide is a platelet GPIIb/IIIa receptor inhibitor that prohibits clot development in a predictable and easy controllable way. Eptifibatide is used during percutaneous coronary intervention (PCI) at a dose of 180 μg/kg twice as bolus injections, followed by 2 μg/kg/min continuous infusion for up to 72h, with a reported bleeding frequency of 2% [Dong et al. 2010]. It was recently demonstrated that platelets play a crucial role in maintaining vascular integrity and preventing hemorrhage in conditions with inflammation [Goerge-T et al. 2008]. Importantly, this protective function was independent of adhesion and aggregation, including blockade of the GPIIb/IIIa receptor, indicating that it was attributed to soluble mediators released from platelets acting in a paracrine fashion [Goerge-T et al. 2008]; a paracrine function that remains intact despite treatment with GPIIb/IIIa receptor blockers like eptifibatide [Xiao et al. 1999]. Furthermore, pooled data from three randomized, placebo-controlled trials evaluating eptifibatide, analyzed for effect during PCI, revealed that thrombocytopenic patients receiving prophylactic eptifibatide had fewer ischemic events (7% vs. 23%, p=0.06) and lower 30-day mortality (4% vs. 15%, p=0.05) compared to placebo treated thrombocytopenic patients. Furthermore, thrombocytopenic patients who received eptifibatide before PCI had fewer subsequent bleeding and transfusion events compared to thrombocytopenic patients receiving placebo [Kereiakes et al. 2000], together supporting continued platelet-function protecting against bleeding despite GPIIb/IIIa receptor blockade.

From a safety point of view the iloprost and eptifibatide doses selected for this trial are lower than the recommended doses for their respective approved indications. The dosages chosen for the current trial are in alignment with doses that have been reported to result in the desired effect for each agent without causing significant adverse side effects.

The safety of the co-administration of eptifibatide and iloprost in a dose similar to the chosen one is supported by an completed Phase I/II trial in patients undergoing primary PCI due to ST-elevated myocardial infarct (eptifibatide 0.5 μg/kg/min + iloprost 1.0 ng/kg/min infused for 24h) [Holmvang et al. 2012] . In this trial no bleeding-related adverse events were reported and no treatment-related adverse events occurred.

3.3 Design rationale

3.3.1 Iloprost dose rationale – safety and efficacy

The dose of iloprost for this trial is 1.0 ng/kg/min continuous infusion for 48h. Intravenous doses of prostacyclin 0.5-2.0 ng/kg/min have been reported to be successful at achieving endothelial modulating/preserving effect with no significant hemodynamic or platelet aggregation complications [Grände et al. 2000, Naredi et al. 2001, Gatward et al. 2008, Fabbri et al. 2009]. The chosen dose of 1.0 ng/kg/min demonstrated beneficial effects on vascular integrity in critically ill patients when administered for 3 days [Naredi et al. 2001] and in patients undergoing Whipple surgery due to pancreatic cancer [Johansson and Ostrowski, unpublished data]. The 1.0 ng/kg/min dose is approximately 5 to 10-fold higher than the normal endogenous production of prostacyclin from the healthy endothelium [Davies and Hagen 1993], a dose expected to restore vascular integrity in septic patients where endothelial injury and dysfunction is expected.

3.3.2 Eptifibatide dose rationale – safety and efficacy
The dose of eptifibatide for this trial is 0.50 µg/kg/min continuous infusion for 48h. This dose has been chosen based on a previously published report by Link et al., demonstrating that administration of a platelet GPIIb/IIIa receptor inhibitor (Tirofiban®) at a bolus dose of 50% and a continuous infusion rate of 25% of the dose recommended for PCI use, in combination with unfractionated heparin for 96 hours, was tolerated in patients with cardiogenic shock and need for dialysis [Link et al. 2008]. Importantly, in this study, treatment with the GPIIb/IIIa receptor inhibitor Tirofiban® was not associated with increased bleeding but was rather associated with a significantly lower number of platelet transfusions and a higher, maintained platelet count, as compared to controls anticoagulated with heparin alone [Link et al. 2008]. The dose of eptifibatide administered in the present study will be 25% of that employed as maintenance dose during PCI, and it will be without a bolus injection, which will limit the bleeding risk.

3.3.3 Use of placebo rationale
The trial uses 0.9% saline as placebo, given in the same amount as the active study drug (two i.v. infusions with volume comparable to the active drug). The use of placebo allows double blinding, which is state-of-the-art in clinical trials to obtain a best possible evaluation of efficacy and safety.

3.4 Investigational products
Investigators should be familiar with the product specifications for the study drugs.

3.3.1 Ilomedin® iloprost, prostacyclin analogue
Ilomedin® (iloprost) is a stable analogue of prostacyclin approved for marketing. For a full description of the clinical and non-clinical results see Appendix 1 for the product specification. Iloprost is approved for treatment of patients with primary pulmonary hypertension, classified as NYHA functional class III, and for treatment of patients with severe thrombangitis obliterans (mb. Bürger).

Summary of results from relevant non-clinical and clinical trials
Acute Hemodynamic Effects: Acute intravenous infusions of Iloprost for up to 15 minutes in patients with secondary and primary pulmonary hypertension produce dose-related increases in cardiac index (CI) and stroke volume (SV) and dose-related decreases in pulmonary vascular resistance (PVR), total pulmonary resistance (TPR), and mean systemic arterial pressure (SAPm). The effects of Iloprost on mean pulmonary artery pressure (PAPm) were variable and minor. In the dose administered in the present trial, no negative effect on blood pressure, central venous pressure or systemic vascular resistance can be measured [Johansson et al. unpublished data].

3.3.2 Integrilin® eptifibatide, platelet GPIIb/IIIa receptor inhibitor
Integrilin® (eptifibatide) is a synthetic cyclic heptapeptide approved for marketing. For a full description of the clinical and non-clinical results see Appendix 2 for the product specification. Eptifibatide is indicated for the prevention of early myocardial infarction in adults presenting with unstable angina or non-Q-wave myocardial infarction, and it is used as an adjunct therapy to aspirin, heparin and ADP-receptor inhibitor and administered as continuous infusion for up to 96 hours.

Summary of results from relevant non-clinical and clinical trials
The pivotal clinical trial for Unstable Angina (UA)/Non-Q Wave Myocardial Infarction (NQMI) was PURSUIT. This study was a 726-center, 27-country, double-blinded, randomized, placebo-controlled study in 10,948 patients presenting with UA or NQMI. Compared to placebo, eptifibatide significantly reduced mortality.

4. Trial objectives

4.1 Objectives
Evaluating the safety and efficacy of co-administration of eptifibatide and iloprost as compared to standard care (placebo) in patients with septic shock.

4.2 Hypothesis

The combination therapy is expected to deactivate the endothelium and restore vascular integrity (iloprost), reduce formation of microvascular thrombosis and dissolve existing clots in the microcirculation (eptifibatide, iloprost), maintain platelet counts (eptifibatide, iloprost) thereby improving platelet-mediated immune function and reducing bleeding. This will translate into reduced organ failure and improved outcome in patients with severe sepsis/septic shock.

5. Trial design

This is a single center, randomized (2:1, active:placebo), placebo controlled, double-blind investigator-initiated phase IIa trial in patients with septic shock, investigating the safety and efficacy of co-administration of Iloprost and Eptifibatide in a total of 18 patients.

12 + 6 patients will be enrolled:

- The active treatment (n=12 patients) will consist of continuous co-administration of i.v infusions of 1.0 ng/kg/min of iloprost for 48 hours and 0.50 µg/kg/min eptifibatide for 48 hours.
- Patients in the placebo group (n=6 patients) will receive double dummy saline infusions and will be treated exactly as active patients.

All patients will be treated according to normal standard, including LMWH thrombosis prophylaxis, which will be initiated in all patients according to standard ICU care.

Patients are presented at the investigator site in a critical acute condition therefore scientific guardians will co-sign the informed consent form. Next-of-kin and the patients' general practitioner will co-sign as soon as possible.
During the study blood samples will be taken at different time points. Patients will be observed and assessed continuously with regards to bleeding. Patients will be actively assessed as long as he/she is in the ICU. During the extended follow up period at day 30 and 90, contact will be made with the patients and/or department to follow up on safety events and establish potential mortality.

5.1 Trial flow diagram

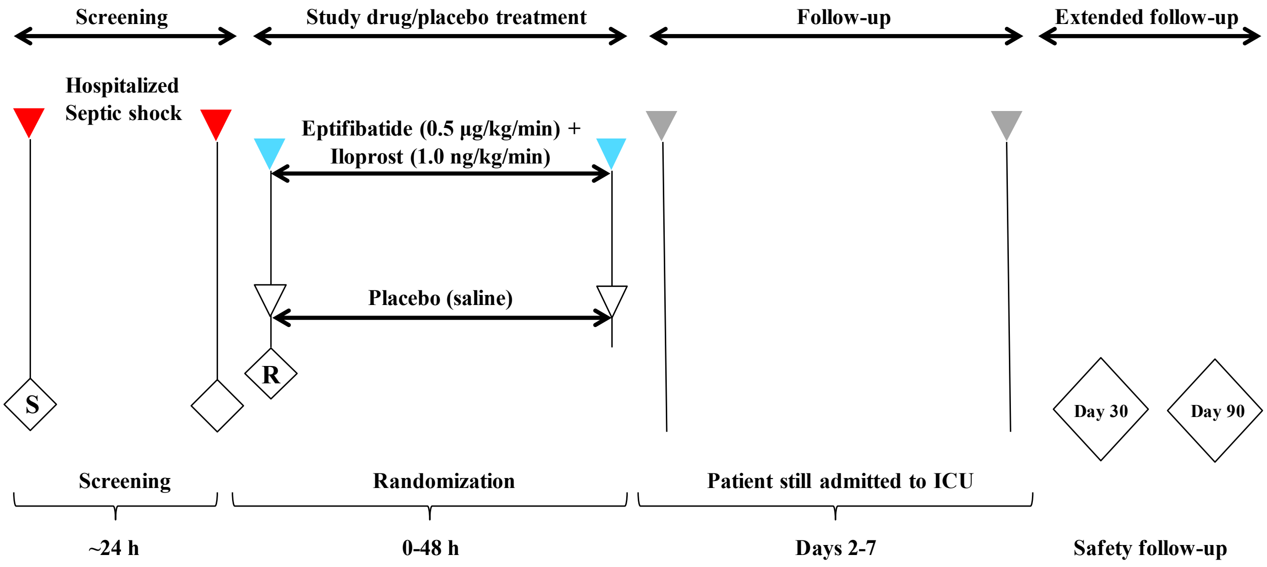

5.2 Trial table of observations and blood sampling

	Screen	Time-point						In case of withdrawal	30d /90d follow-up
**Day**	**pre-drug**	**d1**	**d1**	**d2**	**d3**	**d5**	**d7**		
**Hours**	**0h**	**6h**	**24h**	**48h**	**72h**	**120h**	**168h**		
Informed consent	X								
Demographics/medical history	X								
Height/weight	X								
SOFA scoring[1]	X	X	X	X	X	X	X	X	
Maximum p-lactate	X	X	X	X	X	X	X	X	
AE/SAE and co-medication[2], incl. fluids/blood		Continuously throughout study							
Hematology/biochemistry[3]	X	X	X	X	X	X	X	X	
Endothelial markers[4]	X	X	X	X	X	X		X	
Drug/placebo administration		X	X	X					

[1]SOFA scores include oxygenation ratio, lactate, blood pressure and pulse at pre-study drug infusion, 30 min, 1h, 6h, 12h, 24h
[2]Only concomitant medication affecting the hemostasis will be registered
[3]Blood samples for hematology/biochemistry are taken pre study-drug adm., post 6h, 24h, 48 h, 72h (3d), 120h (5d) and 168h (7d).
[4]Blood samples for endothelial markers and fibrinolysis are taken pre study-drug adm., post 6h, 24h, 48 h, 72 h (3d) and 120h

5.3 Endpoints

5.1.1 Primary endpoints

- Change in biomarkers indicative of endothelial activation and damage (sE-selectin, syndecan-1, thrombomodulin, sVE-cadherin, nucleosomes) from baseline to 48 hours post-randomization
- Change in platelet count from baseline to 48 hours post-randomization
- Change in D-dimer and fibrin split products indicative of fibrinolysis (fibrinogen degradation Bβ15-42; fibrin degradation Fragments X, Y, D and E) from baseline to 48 hours post-randomization

The reason for having 3 primary sub-endpoints is that they reflect different effects of active treatment vs. placebo on the vascular system that we wish to evaluate i.e., endothelial activation, platelet consumption and fibrinolysis.

5.1.2 Secondary endpoints

- Severe bleeding (intracranial or clinical bleeding with the use of 3 RBC units or more/24 hours)
- Use of blood products (in ICU) post-randomization
- Difference in day 7, 30 and 90 day mortality between patients receiving active treatment (eptifibatide and iloprost) and placebo
- Changes in SOFA score from baseline to 48 h and day 5 and 7 post-randomization
- Days of vasopressor, ventilator and renal replacement therapy post-randomization

6. Patient selection and withdrawal

The trial population is adult patients admitted to the ICU with septic shock within the last 24h without having a medical history or condition that specifically increases the risk of bleeding or thrombosis.

6.1 Inclusion criteria

1. Adult intensive care patients (age ≥18 years)
   AND
2. Sepsis, defined as suspected or confirmed site of infection or positive blood culture and ≥2 of 4 systemic inflammatory response syndrome (SIRS) criteria fulfilled within the last 24h:
   a) Temperature ≤ 36˚ C or ≥ 38˚C
   b) Heart rate ≥ 90 beats per minute
   c) Mechanical ventilation for acute respiratory process or respiratory rate ≥ 20 breaths per minute or $PaCO_2 < 4.2$ kPa
   d) WBC ≥ 12,000/mm³ OR ≤ 4,000/mm³ OR > 10% bands
   AND
3. Septic shock within the last 24h, defined as:
   a. Hypotension (MAP <70 mmHg, Lactate 4 mmol/L) despite ongoing resuscitation with fluids (crystalloids, colloids, blood products) within the last 24h OR

   b. ≥30 ml/kg ideal body weight (IBW) fluid (crystalloids, colloids, blood products) given in the last 24h AND
   c. Need for vasopressor/inotropic agents (noradrenaline, adrenaline, dopamine) within the last 24h

   AND
4. Can be randomized into trial and dosed < 24h after septic shock diagnosis (the time-point for the septic shock diagnosis corresponds to the time-point where the vasopressor/inotropic therapy (3c) is initiated)
   AND
5. Consent is obtainable

6.2 Exclusion criteria

Patients are not eligible for inclusion in this trial if they fulfill one or more of the following criteria:

1. Patient is pregnant or breast-feeding
2. Patient weights more than 125 kg
3. Patients with known allergy towards any of the investigational products or contraindications which should be excluded according to the investigational product specifications
4. Patients in whom the clinician finds antithrombotic therapy contraindicated - prophylaxis included
5. Patients at increased risk of bleeding:
   a. Surgery in the previous 48h and expected surgery within 48 h
   b. Epidural or spinal puncture in the previous 12h
   c. Platelet count less than 10,000/mm^3 in the previous 24h
   d. Need of blood products for bleeding in the previous 24h (3 or more RBC/24 h)
   e. Treatment with any antithrombotics within 12h (profylaxis excepted)
   f. Current intracranial bleeding
   g. Traumatic brain or spinal injury within the last month
6. Patients requiring any form of antithrombotics (beyond profylaxis) in therapeutic doses or prothrombotics in any dose, including:
   a. Unfractionated heparin within 8h before the infusion (prophylactic heparin up to 15,000 U/day permitted)
   b. LMWH within 12h before the infusion (prophylactic doses permitted)
   c. Warfarin within 1 day before the infusion
   d. Acetylsalicylic acid more than 650 mg/day within 3 days before the study
   e. Thrombolytic therapy within 3 days before the study (catheter clearance doses permitted)
   f. GPIIb/IIIa receptor inhibitors within 4 days before the study
   g. Antithrombin III with dose greater than 10,000 U within 12h before the study
7. Patients with a do-not-resuscitate order (expected not to survive more than few days because of uncorrectable medical or surgical condition other than sepsis)
8. Patient with chronic renal failure requiring dialysis (renal failure without need for dialysis permitted)
9. Patients who have undergone transplantation of bone marrow, liver, pancreas, heart, lung, or bowel (kidney transplant permitted)
10. Patient with known hypercoagulable condition:
    a. Activated protein C resistance
    b. Hereditary protein C, protein S, or antithrombin III deficiency
    c. Anticardiolipin or antiphospholipid antibody
    d. Lupus anticoagulant
    e. Homocysteinemia
    f. Recent or highly suspected pulmonary embolism or deep venous thrombosis (within 3 months)
11. Patients with known congenital hypocoagulable diseases
12. Patient with known primary pulmonary hypertension

6.3 Removal of patients from the trial, therapy or assessment

If there is medical reason for the withdrawal, the patient should be followed medically until the condition has either resolved itself or is stable and the individual concerned is able to resume care by his/her physician.

Details of the reason for withdrawal should be recorded in the patient's Case Report Form and follow-up data should be entered.
All patients who have received eptifibatide and/or iloprost should continue to be included in the assessments of its safety and efficacy. Patients who are withdrawn should, if possible, have a follow-up examination, including a physical examination, the appropriate investigations, vital signs, and clinical laboratory tests. All details of this follow-up examination should be recorded in the patient's medical source documents.

6.4 Patient withdrawal
Participation in the trial is strictly voluntary. Patients, relatives or scientific guardians are free to discontinue the trial at any time without giving their reason(s).

A patient must be withdrawn from the trial treatment in the event of any of the following:

- Withdrawal of consent
- There are stopping rules as defined in Section 8.8 (serious adverse reactions (allergic reactions, severe bleeding [intracranial or bleeding with need of 5 RBC units or more], severe hypotension, severe hypoxia, SUSAR, clinically relevant thrombosis) which is clinically relevant and affects the patient's safety, and discontinuation is considered necessary by the study investigators
- Intake/administration of prohibited concomitant medication as defined in Section 6.2 where the predefined consequence is withdrawal from the trial
- Intake/administration of the following medication (K-vitamin antagonist treatment, antithrombotics (other than prophylactic LMWH in appropriate patients), prothrombotics, antithrombin III, pro-fibrinolytic drugs, ilomedin/ilporost if not study drug)

All patients who withdraw from the trial for any reason and at any time should have an end of trial examination. Patients will be examined for any status changes that require further follow-up. All withdrawn patients will be followed-up as the remaining patients in the trial. If consent is withdrawn, the person making the withdrawal will be asked for permission to follow-up for 90 days after randomization.

6.5 Trial discontinuation
The whole trial may be discontinued at the discretion of the principal investigator in the event of any of the following:

- Medical or ethical reasons affecting the continued performance of the trial
- Difficulties in the recruitment of patients

7. Trial treatment

7.1 Identity of the investigational product
Two marketed products will be administered in this trial, iloprost and eptifibatide.
Ilomedin®, iloprost or prostacyclin, is used to treat primary pulmonary hypertension.
Integrilin® eptifibatide or platelet GPIIb/IIIa receptor inhibitor, is an antithrombotic agent used in the prevention of myocardial infarction.

7.1.1 Packaging and labeling of the investigational product
The drug used in this trial will be labeled according to local regulations.

7.1.2 Storage, issue, and return of investigational product
The medication is paid and delivered by the Sponsor (Sisse R. Ostrowski) to the trial site before commencement of the trial. A copy of a signed receipt will be kept in the trial files. After the trial is completed, the principal investigator should be contacted to determine how to treat any leftover medication. The Investigator will be responsible for the storage, dispensing, inventory, and accountability of all clinical supplies. An accurate, timely record of the disposition of all clinical supplies must be maintained as described below:

- The identification of the patient to whom the drug was dispensed
- The date(s) and quantity of the drug dispensed to the patient
- The product lot number

The preparation of the Study Drugs must be documented on a 'Drug Preparation and Dispensing Log Form' filed in the pharmacy site file.

7.2 Description of the investigational product

7.2.1 Iloprost

Iloprost is a marketed product, a description of it can be found in the product specification in Appendix 1. It will be handled as described.

7.2.2 Eptifibatide

Eptifibatide is a marketed product, a description of it can be found in the product specification in Appendix 2. It will be handled as described.

7.3 Randomization

Patients will be randomized to receive either active treatment or placebo.
Code envelopes (12 active treatment, 6 placebo) are made by the principal investigator, and delivered to the clinical trial site. Randomization lists and code envelopes will be filled out and handled by a study nurse at the perioperative section (opvågningsafsnit), dept. of anesthesia, Nordsjællands Hospital. A randomization list will be held by Kai Lange (Chief physician, DMSc, DESA, dept. of anesthesia, Nordsjællands Hospital) which will be available to the investigator after the completion of the trial.
Emergency code breaking envelopes will be available on-site (at Kai Langes office) 24/7 for the treating investigators/personal.

7.4 Preparation of trial study drug (active, placebo)

The trial drug will be prepared as described in the product specifications, Appendix 1 and 2, in brief described below.
Medical charts encompassing information about delivery of study drugs to the trial site will be created along with individual medical charts encompassing information about the study drugs administered to the individual patient. In addition to this, the study drug infusions will be noted in the standard medical chart.
The preparation of the study drug will be done by an unblinded study nurse from the perioperative section (opvågningsafsnit), dept. of anesthesia, Nordsjællands Hospital, who is not otherwise involved in the trial and who will be responsible for preparing the study drug so that it can be administered in double-blinded fashion. The correctness of study drug preparation in relation to the randomization code, both at the first and second preparation (at 0h and 24h), will be double checked by an independent nurse from the perioperative section.

Brief description of study drug preparation

Iloprost and eptifibatide are colorless fluids that are to be diluted in 0.9% saline.
The saline 0.9% bags containing diluted active drug/placebo will not be wrapped or sealed to hide the content as there is no difference between how the fluid looks and behaves in the active drug and placebo saline bags.
The following dilutions are done and administered:

7.4.1 Iloprost (study drug 1)

Delivered in 1 ml (20 µg/ml) vials.
Syringe pump: 1-10 ml iloprost (20 µg/ml) is diluted in 10-100 ml 0.9% saline to a final concentration of 2 µg/ml iloprost (a 1:10 dilution). To administer 1 ng/kg/min iloprost, the infusion rate in ml/h for patients with a body weight from 40-120 kg will be:

Weight	Infusion rate (ml/h)	Iloprost vials	Dilution	Dilution vol.	24h infusion vol.
40 kg	1.2	3	3 ml iloprost + 27 ml saline	30 ml	28.8
50 kg	1.5	4	4 ml iloprost + 36 ml saline	40 ml	36.0
60 kg	1.8	5	5 ml iloprost + 45 ml saline	50 ml	43.2
70 kg	2.1	6	6 ml iloprost + 54 ml saline	60 ml	50.4
80 kg	2.4	6	6 ml iloprost + 54 ml saline	60 ml	57.6
90 kg	2.7	7	7 ml iloprost + 63 ml saline	70 ml	64.8
100 kg	3.0	8	8 ml iloprost + 72 ml saline	80 ml	72.0
110 kg	3.3	8	8 ml iloprost + 72 ml saline	80 ml	79.2
120 kg	3.6	9	9 ml iloprost + 81 ml saline	90 ml	86.4

7.4.2 Eptifibatide (study drug 2)
Delivered in 10 ml (2 mg/ml) vials.
10-50 ml eptifibatide (2 mg/ml) is diluted in 10-50 ml 0.9% saline to a final concentration of 1 mg/ml eptifibatide (a 1:2 dilution). To administer 0.5 µg/kg/min eptifibatide, the infusion rate in ml/h for patients with a body weight from 40-120 kg will be:

Weight	Infusion rate (ml/h)	Eptifibatide vials	Dilution	Dilution vol.	24h infusion vol.
40 kg	1.2	2	20 ml eptifibatide + 20 ml saline	40 ml	28.8
50 kg	1.5	2	20 ml eptifibatide + 20 ml saline	40 ml	36.0
60 kg	1.8	3	30 ml eptifibatide + 30 ml saline	60 ml	43.2
70 kg	2.1	3	30 ml eptifibatide + 30 ml saline	60 ml	50.4
80 kg	2.4	3	30 ml eptifibatide + 30 ml saline	60 ml	57.6
90 kg	2.7	4	40 ml eptifibatide + 40 ml saline	80 ml	64.8
100 kg	3.0	4	40 ml eptifibatide + 40 ml saline	80 ml	72.0
110 kg	3.3	4	40 ml eptifibatide + 40 ml saline	80 ml	79.2
120 kg	3.6	5	50 ml eptifibatide + 50 ml saline	100 ml	86.4

7.4.3 Placebo (study drug 1)
0.9% saline, volume ranging from 30-100 ml per 24h.
The precise volume and infusion rate to be administered to the patient is similar to the volume calculated for both of the active drugs.

7.4.4 Placebo (study drug 2)
0.9% saline, volume ranging from 30-100 ml per 24h.
The precise volume and infusion rate to be administered to the patient is similar to the volume calculated for both of the active drugs.

After dilution, iloprost and eptifibatide can last for 24h meaning that a nurse has to prepare an appropriate amount of the drugs (active, placebo) two times: Immediately after randomization (for infusion the first 24h) and again after 24h (for infusion the last 24h).
After preparation/dilution of the study drugs (active, placebo), a pre-formed label is put on each of the two bags and filled out with preparation date and time, expiry time-point, infusion rate and initials of the nurse.

The labels design will be as follows:

Active drug (2 µg/ml iloprost) or placebo (0.9% saline)	Active drug (1 mg/ml eptifibatide) or placebo (0.9% saline)
__________ ml	__________ ml
Prepared (dd-mm-yy, hh:mm): __________<br>Expiry (24h from preparation)<br>(dd-mm-yy, hh:mm): __________	Prepared (dd-mm-yy, hh:mm): __________<br>Expiry (24h from preparation)<br>(dd-mm-yy, hh:mm): __________
**Infusion rate: __________ ml/h**	**Infusion rate: __________ ml/h**
Initials: __________	Initials: __________

The study drug will not be marked with information from Appendix 1-2 since the patients will never administer the study drug to themselves.

7.5 Dosage and administration of study drug
All patients will receive 48 hour continuous infusion of either active study drug or placebo.
Patients on active treatment will receive continuous infusion of 0.50 µg/min/kg eptifibatide and 1.0 ng/kg/min iloprost.
The infusion volume of the active study drug and placebo will be in the range 60-200 ml per 24h, depending on the weight of the patient.

7.6 Medications causing withdrawal of patients during trial

- K-vitamin antagonist treatment
- Antithrombotics (other than prophylactic LMWH in appropriate patients)
- Prothrombotics
- Antithrombin III
- Pro-fibrinolytic drugs
- Ilomedin if not study drug

7.7 Treatment compliance

The Investigator will record the time and dose of administrations of drug affecting coagulation in the medical source documents. Any reasons for non-compliance will also be documented, including:

- Interruptions in the schedule of administration
- Non-permitted medications (Section 7.6)

8. Trial schedule of events

8.1 Patient eligibility

For trial entry, all patients must fulfill all the inclusion criteria, described under Section 6.1. No patient with any exclusion criterion from the list described in Section 6.2 may participate. To assure compliance with the entry criteria, all the assessments described in Section 9.1 to 9.3 must be completed at screening to determine the patients' eligibility for entry into the trial.
All patients subjected to screening will receive a Screening Number and should be listed on the Patient Screening and Allocation Log. The reasons for not entering the study should be provided for patients failing screening.
The Patient Screening and Allocation Log will as a minimum contain:

- Date of Birth
- Screening Number (if applicable)
- Patient Number (if applicable)
- Reason for failing screening (if applicable)

8.2 Patient information/Informed consent procedure

As the patients eligible for this trial are unable to consent because of acutely illness, two scientific guardians, who are independent of the trial, will be asked to consent on behalf of the patient, and sign the consent form. The Investigator must as soon as possible obtain consent from the next-of-kin, the patients' general practitioner and the patient. They will all be given sufficient time to read the trial information and consider participation. If the next-of-kin, the patients' general practitioner and the patient accept participation and sign the informed consent the trial procedures will continue as planned.
One copy of the informed consent will be given to the patient and the original will be documented in the medical source documents.
In cases where the next-of-kin, the patients' general practitioner or the patient will not consent, trial-related procedures will stop, but the data obtained until then will be used in the final database unless the patient wants data deleted.

8.3 Treatment, observation and follow up

The investigator must have evaluated the patient's eligibility and approved the patient's enrollment in the study prior to administering study drug/placebo. The study/placebo drug is administered continuously i.v. for 48 hours. The Investigator will during the infusion observe the patient especially with regards to potential signs of bleeding.
Blood samples for hematology/biochemistry and endothelial biomarkers will be drawn pre-study drug/placebo administration, post 6h, 24h, 48h and 72 h, 120h and 168h according to Section 5.2.
AE and SAE and changes in concomitant medication affecting the coagulation and procedures will be reported continuously if applicable and noted in the patient records as well as the CRF.

8.4 Extended follow-up

Extended follow up is not part of the main study. Patients will be followed for up to 90 days. Beyond that patients will only be followed up with regards to safety. Any on-going adverse reactions will be followed up including mortality.

8.5 Recruitment period

First patient in: September 2014
Last patient in: August 2015

8.6 Number of patients

A total 18 evaluable patients will be recruited in a 2:1 ratio active:placebo.
The number of patients participating is not based on power calculation as there is no available data on the specific active drug dual-therapy used in the present study vs. placebo. However, based on data from previously conducted trials evaluating safety and efficacy of prostacyclin, the chosen number of patients (12 + 6) is expected to be able to show trends (see power calculation in Section 11.4). Patients whom drop out or are withdrawn for any reason before Day 7 will be replaced.

8.7 Treatment allocation

The trial groups will be completed.

8.8 Stopping rules

- Serious adverse reactions: allergic reactions, severe bleeding (intracranial or bleeding with need of 5 RBC units or more), severe hypotension, severe hypoxia
- SUSAR
- Clinically relevant thrombosis

9. Trial assessments

The following sections describe the methods of assessments and list the type of data to be recorded in the case report form (CRF).

9.1 Clinical assessments

9.1.1 Demographic data, medical history, and physical examination

A medical history (for details see below), including demographics must be completed prior to study drug/placebo dosing. Data to be collected at the screening examination (noted in the patient file) are as listed below:

- Relevant medical history
- Prior bleeding tendency e.g. vW-disease, hemophilia, prior thrombotic events, thrombocytopenia or thrombocytopathy
- Time of septic shock diagnosis
- Relevant concomitant diseases and treatments
- Relevant concomitant medications (route, dose, frequency)
- Pregnancy test results are to be documented (serum or urine testing is acceptable)
- Data for SAPS, SOFA and APACHE

A report of the examinations is to be made available in the patient's source documents for the trial.

9.1.2 Vital signs and weight

A normal physical examination will be performed that includes weight, height, SOFA sub-scores and p-lactate and arterial blood gasses.

9.2 Laboratory assessments

The normal procedures for sampling, handling, storage, and transfer of the laboratory samples will be followed. All material such as test tubes and labels will be labeled with the patient number and study number in addition to the routinely used information. The reference ranges used are those of the local laboratory at the trial site.

9.2.1 Biochemistry and hematology
Blood samples will be drawn pre-study drug/placebo administration, post 6h, 24h, 48h, 72 h, 120h and 168h. Since routine blood samples are drawn at least once daily in patients while in the ICU, the 6h sample is the only blood sample that exceeds routine blood samples.
All samples will be transferred to the local laboratory at trial site for analysis of the following parameters:

- Arterial blood gas (pH, PaO2, PaCO2, HCO3-, Ca2+, lactate), hemoglobin, leukocyte count, platelet count, bilirubin*, BUN, creatinine, sodium*, potassium*, albumin, CRP, ALAT*, ALP (Alkaline Phosphatase)*, INR, AT, APTT, D-dimer, PCT

*Will not be taken at 6h
Blood tubes required for the above analysis:

- 4 ml EDTA tube
- 4 ml citrate tube

9.2.2 Endothelial and fibrinolysis biomarkers
Blood samples will be drawn pre-study drug/placebo administration, post 6h, 24h, 48h, 72h and 120h. All samples will be transferred to the local Blood Bank at the trial site for further processing (centrifugation, plasma isolation), aliquoting and freezing.
The Blood Bank Hemostasis laboratory, 2034, Rigshospitalet, will analyze the plasma samples taken during the trial for the endothelial and fibrinolysis biomarkers. The samples will be stored and analyzed at one time-point at the end of trial. Because of cost, all markers may not be analyzed.
The following markers are planned to be measured pre-study drug/placebo administration, post 6h, 24h, 48h, 72h, 120h:

- sE-selectin, syndecan-1, thrombomodulin, sVE-cadherin, histone-complexed DNA fragments, Bβ15-42, Fragments X, Y, D and E

Blood tubes required for the above analysis:

- 4 ml EDTA tube
- 4 ml citrate tube
- 4 ml serum tube

9.2.4 Research biobank
In this study, the blood samples taken will be processed and stored frozen as plasma at a laboratory (initially at the local Blood bank, later in the Blood bank at Rigshospitalet) before being analyzed at the end of the study. The purpose of this biobank is to examine if the active drug compared to placebo has effects on the vascular system (endothelium, fibrinolysis) and other aspects of the disease.
The collected plasma samples will be stored at the laboratory in the Blood bank at Rigshospitalet in Denmark and all samples will be analyzed upon completion of the study. After completion of the biomarker analysis the plasma samples will be destroyed. The biological material taken will not be transferred to other countries. At every blood sampling approximately 15 ml will be taken, which in total will be 90 ml for the whole study.

9.3 The CRF
Data on demography, anthropometry, clinical status, biochemistry/hematology and standard therapeutic interventions will be collected from baseline (pre-intervention). Data on clinical status, biochemistry/hematology and standard therapeutic interventions will be collected during the intervention (at 6h, 24h and 48h) and after the intervention (3d, 5d and 7d after inclusion). Data on drop-out, withdrawal, death will be collected along with data on ARs/AEs/SAEs/SARs/SUSARs.
The only data entered directly into the CRF are: Inclusion date and time, time-point for septic shock diagnosis, if active treatment/placebo was prematurely ceased (and causes for this i.e., inevitably dying patient), study completion and causes for not completing the study (drop-out, withdrawal, death), drop-out/withdrawal causes (AE/AR/SAE/SAR/SUSAR) and AE/AR/SAE/SAR/SUSAR.

10. Serious adverse reactions
The serious adverse reactions (SARs) described with the use of the trial drugs are as follows:
Iloprost (Ilomedin®)

Very common (>10%)	Headache, flushing, nausea, vomiting, hyperhidrosis

Common (1-10%)	Pyrexia/increased body temperature, reduced appetite, fatigue/tiredness, thirst, uncomfortably feeling, pain, infusion site reactions, abdominal discomfort/pain, diarrhea, tachy-/bradycardia, angina pectoris, hypo-/hypertension, dyspnea, arthralgia, myalgia, apathy, confusion, hyper-aesthesia, paresthesia, trismus, restlessness, agitation, sedation, vertigo
Not common (0.1-1%)	Dysphagia, rectal hemorrhage, dry mouth, jaundice, arrhythmia, myocardial infarction, cardiac failure, cerebral ischemia, deep vein thrombosis, pulmonary edema, pulmonary embolism, asthma, thrombocytopenia, muscle spasms, migraine, increased muscle tonus, anxiety, depression, hallucination, pruritus, hypersensitivity, kidney pain, urinary tract infection, dysuria, blurred vision, eye irritation, eye pain

Source:
http://pro.medicin.dk/Medicin/Praeparater/1506
Summaries of Product Characteristics (SPC) section 4.8 (Appendix 1)

Eptifibatide (Integrilin®)

Very common (>10%)	Bleeding tendency
Common (1-10%)	Ventricular fibrillation/tachycardia, AV-block, thrombophlebitis
Not common (0.1-1%)	Cerebral ischemia, thrombocytopenia
Rare (0.01-0.1%)	Hypotension
Very rare (< 0.01%)	Infusion site reactions, anaphylactic reaction

Source:
http://pro.medicin.dk/Medicin/Praeparater/2578
Summaries of Product Characteristics (SPC) section 4.8 (Appendix 2)

10.1 Assessment and registration of SARs/SAEs and SUSARs

The most serious adverse reactions described with the use of the study drugs are allergic reactions, thrombosis, bleeding and thrombocytopenia. The occurrence of these AR/SARs will be looked after and recorded daily in the CRF during the ICU stay and requested as part of the follow up data.
Specifically, the following data are collected in the CRF:
Routine biochemistry/hematology variables (pre-study drug/placebo administration, post 6h, 24h, 48h, 72h and 120h): Platelet count (thrombocytopenia), D-dimer (thrombosis), hemoglobin (bleeding)
Clinical data: Occurrence of serious bleeding / transfusion requirement (CT verified intra cranial hemorrhage, fresh upper/lower gastrointestinal bleeding, other bleeding (requiring or not requiring surgery), use of RBC in the last 24h)
ARs/AEs and SUSARs/SARs/SAEs: Allergic reaction

10.2 Reporting of SARs/SAEs and SUSARs

SARs will be reported immediately to the sponsor to determine if this is a SUSAR.
Up until day 30, AR and SARs will be compared between placebo and active treatment.
The GCP-unit will be given access to blinded data in the CRF to enable continuous monitoring.
During the trial, the Investigator will send a yearly report on the occurrence of SARs to the Danish Health and Medicines Authority and Ethics committee. Since the trial is planned to last 1 year, the 1 year report on the occurrence of SARs/SAEs will represent the final report on this topic to be sent to the Danish Health and Medicines Authority and Ethics committee.

Suspected unexpected serious adverse reactions (SUSARs) will be defined as serious adverse reactions not described in the SPC for iloprost and eptifibatide. SUSARs will be reported according to regulation directly to the Danish Health and Medicines Authority and Ethics committee.

The sponsor must ensure that all relevant information about SUSARs, which are fatal or life-threatening, is recorded and reported to the Danish Health and Medicines Authority and Ethics committee as soon as

possible and no later than 7 days after the sponsor is informed of such a SUSAR. No later than 8 days after the reporting, the sponsor must inform the Danish Health and Medicines Authority and Ethics committee of relevant follow-up information on the sponsor's and the investigator's follow-up action to the reporting. Any other SUSARs must be reported to the Danish Health and Medicines Authority and Ethics committee no later than 15 days from the time when the sponsor is informed about them.

Per definition, patients admitted to the ICU because of critical illness will, with a high likelihood, experience several AE and SAEs. As recording all these will not add valuable information to the patients safety in the this trial, SAEs which affect the SOFA score, thus worsening of cerebral, respiratory, circulatory, hepatic, renal and coagulation failure will be only be captured as this per SOFA score definition changes the score for the specific organ.
Thus the SAEs will be captured in the secondary outcome measures, which will be compared for placebo and active treatment.

11. Analysis of trial data

11.1 Endpoints

11.1.1 Primary endpoints

- Change in biomarkers indicative of endothelial activation and damage (sE-selectin, syndecan-1, thrombomodulin, sVE-cadherin, nucleosomes) from baseline to 48 hours post-randomization
- Change in platelet count from baseline to 48 hours post-randomization
- Change in D-dimer and fibrin split products indicative of fibrinolysis (fibrinogen degradation Bβ15-42; fibrin degradation Fragments X, Y, D and E) from baseline to 48 hours post-randomization

The reason for having 3 primary sub-endpoints is that they reflect different effects of active treatment vs. placebo on the vascular system that we wish to evaluate i.e., endothelial activation, platelet consumption and fibrinolysis.

11.1.2 Secondary endpoints

- Severe bleeding (intracranial or clinical bleeding with the use of 3 RBC units or more/24 hours)
- Use of blood products (in ICU) post-randomization
- Difference in day 7, 30 and 90 day mortality between patients receiving active treatment (eptifibatide and iloprost) and placebo
- Changes in SOFA score from baseline to 48 h and day 5 and 7 post-randomization
- Days of vasopressor, ventilator and renal replacement therapy post-randomization

11.1.3 Other endpoints

In addition, laboratory parameters (biochemistry and hematology), vital signs and maximum p-lactate will be summarized using descriptive statistics.

11.2 Definitions of evaluability

The definitions of study populations are as follows:

Population	Definition
Intention-to-treat	This will comprise all patients who receive any study drug, even if not completed.<br>This population will be evaluable for safety.
Per-protocol	This is a subset of the intention-to-treat population.<br>It includes patients who have received the study drug infusion for 48 hours and who have not been given any prohibited medications.
Evaluable for response	This is a subset of the per-protocol population.<br>It includes patients who received the study drug for at least 24 hours, these patients will be evaluated for an efficacy response.

Number of patients in and available data from all three populations will be described in the required reports to the Danish Health and Medicines Authority and Ethics committee and in peer-reviewed scientific papers.

11.3 Statistical methods

This is a pilot trial and thus all statistical tests should be considered to be exploratory.

Descriptive statistics will be calculated for all endpoints. All summary statistics of continuous variables will include: n, mean with standard deviation, median with min/max and inter quartile ranges. All summary statistics of frequency tables will include n, % and N, where N is the total number of patients recorded values in the corresponding group.
P-values <0.05 are considered significant.

11.3.1 Primary endpoints
The difference between treatment groups for continuous data will be evaluated using analysis of variance (mixed model) followed by post hoc pairwise comparisons of means. Furthermore, delta values (numerical change in variables between time-points) within and between groups will be compared by paired (wilcoxon-signed rank test) and non-paired (Mann-Whitney U test) non-parametric tests.

11.3.2 Secondary endpoints
The difference between treatment groups for categorical data will be evaluated using McNemars test (change over time), frequency tables and chi-square statistics.
The difference between treatment groups for continuous data will be evaluated using analysis of variance (mixed model) followed by post hoc pairwise comparisons of means. Non-parametric test, Wilcoxon rank sum test, will be used if the assumption of normality is not fulfilled.
Mortality will be evaluated applying survival statistics i.e., Kaplan Meier plots and log rank test and Cox proportional hazards models.

11.3.3 Other endpoints
Data will be tabled and summarized descriptively.

11.4 Sample size and power
Patients whom drop out or are withdrawn for any reason before Day 7 will be replaced.
The number of patients participating is not based on power calculation as there is no available data on the specific active drug dual-therapy used in the present study vs. placebo. However, based on data from previously conducted trials evaluating safety and efficacy of prostacyclin in double blinded trials randomizing patients to: prostacyclin vs. placebo (Whipple surgery study, [Johansson et al. unpublished data]) or prostacyclin + eptifibatide (1 ng/kg/min and 0.5 µg/kg/min i.e., same doses as applied in this trial) vs. eptifibatide alone (0.5 µg/kg/min) (PCI patients study, [Holmvang et al. 2011]), the chosen number of patients (12 + 6) is expected to be able to show trends:
<u>Whipple surgery study</u>
In this study, 20 + 20 patients undergoing Whipple surgery were randomized to active drug vs. placebo to investigate the effect of prostacyclin infusion (1 ng/kg/min) on hemostasis and the endothelium. The post-surgery increase in circulating sVE-cadherin differed significantly among the active (mean ±SD 1654 ±422 pg/ml) and placebo (mean ±SD 1978 ±461 pg/ml) group (p=0.035), and to detect this difference 1-sided with a power of 0.60 (1-β) and alpha of 0.05 requires n=15 patients in each group (SAS statistical software).
In an secondary part of this study (abandoning colloid fluids) investigating further 8 + 8 patients, the post-surgery RBC transfusion requirement during study drug infusion (prostacyclin, placebo) differed significantly among the active (mean ±SD 0 ±0 ml RBC) and placebo (mean ±SD 238 ±343 ml RBC) group (p=0.027), and to detect this difference 1-sided with a power of 0.60 (1-β) and alpha of 0.05 requires n=11 patients in each group (SAS statistical software).
<u>PCI patients study</u>
In a study of 8 + 8 patients evaluating the safety and efficacy of prostacyclin + eptifibatide (1 ng/kg/min and 0.5 µg/kg/min) vs. eptifibatide alone (0.5 µg/kg/min), the change in sE-selectin from baseline to 24h after ceasing the study drug infusion differed significantly among the active (decreased -3.40 ng/ml) and placebo (increased +4.26 ng/ml) group (p=0.008).

11.5 Source data and patient files
Most data will be entered into the CRF from patient files (source) by trial or clinical personnel under the supervision of the trial site investigators.
Data entered directly into the CRF are: Inclusion date and time, time-point for septic shock diagnosis, if active treatment/placebo was prematurely ceased (and causes for this i.e., inevitably dying patient), study

completion and causes for not completing the study (drop-out, withdrawal, death), drop-out/withdrawal causes (AE/AR/SAE/SAR/SUSAR) and AE/AR/SAE/SAR/SUSAR.
When all patients have completed their visit and data from 30-day follow-up are collected, the database will be cleaned and locked. Statistical analysis and reporting will be done.
Data from the 90-day follow-up visits will be cleaned, locked and analyzed separately.

12. Ethical considerations

12.1 Independent ethical committee

The study is conducted in accordance with the Helsinki 2 declaration and the protocol, any amendments, the consent form, and the patient information must be approved by the health authorities (the Danish Health and Medicines Authority) and the Danish Data Protection Agency according to Danish legislations and by appropriately constituted independent ethical committee before study initiation. The study is reported to and approved by the Danish Data Protection Agency through the common application form of the Capital Region, Denmark. The study complies with the Danish Health Act (Sundhedsloven).

12.2 Patient information and informed consent

Patients considered to be included in the trial will be temporarily incompetent because of acute, severe illness. To make clinical trials with the goal of improving the treatment of septic shock, a life threatening condition, it is necessary to include unconscious and incompetent patients as no clinically relevant animal model exists and no conscious patients have a disease severity expected to benefit from early treatment with the intervention suggested in this study.

The principal investigator or his/her designee must when possible obtain the written Informed Consent from the scientific guardians, or next of kin and general practitioner. Each patient or next of kin must receive full patient information from the primary investigator or his/her designee (according to the GCP rules), before giving consent. The patient information must contain full and adequate verbal and written information regarding the objective and procedures of the study and the possible risks involved. Patients, who during the course of the study, become able to give consent, will be asked to participate and give their consent.
Before signing the Informed Consent Form, the patient or next of kin must be given sufficient time i.e., >24 hours, to consider possible participation. The patient or next of kin are allowed to bring an assessor when the information about the trial is provided. Furthermore, each patient or next of kin must be informed about the right to withdraw from the study at any time. The information to the patient or next of kin about the trial will be provided by the primary investigator or his/her designee in a quiet undisturbed location i.e., in a private ward or in a private room in the ICU.
Each patient or next of kin must sign the Informed Consent form; the patient receives a copy of the signed form and the original is retained in the Investigator Site File. The Informed Consent forms must be signed and dated both by the patient/next of kin and by the investigator providing the information to the patient.

Patients will only be enrolled after informed consent, but as the treatment has to be initiated earliest possible after the septic shock diagnosis i.e., at a time-point where patients are temporarily incompetent and the next of kin may not have reached the hospital yet and it may be outside opening hours of the general practitioner, it may be impossible to obtain surrogate consent from next of kin and general practitioner. In this situation, patients may be included after proxy consent by two independent physicians (scientific guardians). The scientific guardians will be familiar with the trial protocol, trial subject information and other documents related to the trial before giving their proxy consent.

12.3 Ethical and risk/benefit considerations

The following ethical considerations have been considered for the proposed clinical trial:

Is this trial necessary?
Severe sepsis affects millions of patients worldwide with high rates of complications and mortality. To date, there is no registered drug for severe sepsis/septic shock, so further drug development is important in an attempt to reduce mortality and morbidity from sepsis.

What is the risk/benefit for the participating patients?

Both drugs are already used clinically, so the side effects are known. Furthermore, both drugs are used in considerably lower doses than normally used. Participation carries minimal risk for the patients as those with increased risk of side effects are excluded and there are strict stopping criteria if the risk of side effects increases during trial.

Potential benefit for the participating patients?
The trial is being conducted to improve the treatment of septic shock so it is expected that the health of the trial subjects will improve in the long run.

13. Monitoring and quality assurance (QA)

13.1 Compliance with Good Clinical Practice (GCP), national legislation and quality standards and local SOPs

This Trial Protocol is designed to comply with the Guideline produced by the International Conference on Harmonization (ICH) on the topic Good Clinical Practice (GCP) and published by the European Agency for the Evaluation of Medicinal Products as "Note for Guidance on Good Clinical Practice" (CPMP/ICH/135/95) (Approval 17 July 1996) as well as other relevant guidelines issued by ICH, primarily the efficacy guidelines.
The study will comply with standard procedures for quality assurance & quality control.

13.2 Monitoring

Prior to inclusion of the first patient, investigative site personnel will document experience with GCP, and will receive appropriate training and instructions in the current protocol to enable trial conduct in accordance with GCP.
Monitoring visits to the trial site will be made periodically during the trial, to ensure that all aspects of the protocol are followed. Source documents will be reviewed for verification of agreement with data on Case Report Forms. Also, the trial site may be audited and inspected by appropriate regulatory agencies.
The principal investigator who works a clinical doctor at the trial site has himself direct access to, and guarantees direct access to source data/documents (including patient files) at monitoring, auditing and/or inspecting visits by the GCP-unit and/or the Danish Health and Medicines Authority.
It is important that the Investigator and their relevant personnel are available during the monitoring visits and possible audits and that sufficient time is devoted to the process.

13.3 Source data verification

Source Documents are original documents, data and records (e.g. hospital records, clinical and office charts, laboratory notes, memoranda, x-rays, subject files and records kept at the pharmacy, recorded data from automated instruments etc.). Source Data are considered all information in original records and certified copies of clinical findings, observations, or other activities in the study. Source Data are contained in Source Documents (original records or certified copies).
The location of source document will be registered on a form specifying where source data can be located e.g. medical record, CRF, lab reports etc.
All screening CRFs will be monitored, the monitoring of the rest of CRFs will be about thirty percent of full CRFs. In cases repetitive mistakes are found, this number might increase.

The following items must be available for Source Data Verification (SDV) in source documents other than the CRF:

- Date of conducting Informed Consent
- Date of birth and sex
- Statement that the patient is participating in clinical trial CO-ILEPSS with study drug/s.
- Date for evaluation of eligibility criteria
- Relevant medical history and diagnosis
- Screening Number and Patient Number
- Administration of trial drugs
- All study dates
- Adverse reactions, serious adverse reactions, or absence of these
- Concomitant Medication affecting coagulation

- Date and reason for exclusion or withdrawal

13.4 CRF handling

The main objective is to obtain those data required by the trial protocol in a complete, accurate, legible and timely fashion. The data in the CRFs should be consistent with the relevant source documents.
All data must be stored in an anonymous form in accordance with the data-protection legislations. The study is being reported to the Danish Data Protection Agency.
The CRFs must be suitable for submission to the regulatory authorities.

13.5 Changes to the final protocol

Any variation in procedure from that specified in the Final Trial Protocol may lead to the results of the trial being questioned and in some cases rejected. Any proposed protocol change will be documented in a protocol amendment and this will be submitted to the Ethics Committee and the Regulatory authority for approval.

13.6 Deviations from the trial protocol

Deviations from the trial protocol, especially the prescription of doses not scheduled in the trial protocol, other modes of administration, other indications, and longer treatment periods are not permissible (except in an emergency).

14. Finances

This research project is investigator-initiated by the trial sponsor and co-investigator Sisse R. Ostrowski and co-investigator Pär I. Johansson in collaboration with the principal investigator Morten Bestle. It has not received funding from any commercial sponsors.
The amount paid per patient will be DKK 10,000 in alignment with previous non-industry funded clinical trials in this cohort of patients performed in Denmark. These costs are covered by Pär I. Johansson, Professor, Senior Physicial, DMSC, MPA, The Capital Region Blood Bank, Rigshospitalet. Drs Sisse R. Ostrowski and Pär I. Johansson will apply Danish funds to cover other costs. This process is currently ongoing. The ethical committee and current trial subjects will be informed about grant provider(s) and amount given once they are known.
Expenses related to the trial (expenses to assisting staff, blood samples and laboratory analyses etc.) are held by the involved departments (Blood bank, Rigshospitalet and Dept. of Anesthesia and Intensive Care, Nordsjællands Hospital) as decided upon in the steering committee.
The principal investigator, co-investigators and/or the sponsor is not financially attached to private enterprises, foundations etc.

15. Insurance

The patients in the present study are covered by the patient insurance, covering all treated patients at the trial site (Nordsjællands Hospital, Hillerød (Denmark insurance)) in the event of study related injury or death occurs in accordance with applicable law and with the CPMP Note for Guidance on Good Clinical Practices (CPMP/ICH/135/95) of 17 July 1996.

16. Publication of trial results

The trial will be registered on www.clinicaltrials.gov.
Upon trial completion, manuscript(s) will be published in a peer review clinical journal regardless of the results. The Steering committee will grant authorship depending on personal input according to the Vancouver definitions. The primary manuscript and/or a finalizing report describing the study design, safety (AR, SAR) and efficacy will be sent to the Danish Health and Medicines Authority and Ethics committee within 1 year after completion of the study.
The preliminary listing of authors for the primary manuscript will be as follows: SRO, MB, REB, TSI, PJO. Secondary manuscripts may have other listing of authors as determined by the Steering committee and according to the Vancouver definitions.

17. Trial organization

This trial is investigator-initiated by trial sponsor Sisse R. Ostrowski and Pär I. Johansson, as a collaborative research between the Blood Bank at Rigshospitalet and Department of Anesthesia and Intensive Care, Nordsjællands hospital, both Copenhagen University Hospital.
The project will be managed by the steering committee consisting of: Pär I. Johansson (PJO), Morten Bestle (MB) and Sisse R. Ostrowski (SRO).

18. References

A randomised, blinded, trial of clopidogrel versus aspirin in patients at risk of ischaemic events (CAPRIE). CAPRIE Steering Committee. Lancet 1996; 348:1329-1339

Abbo KM, Dooris M, Glazier S et al. Features and outcome of no-reflow after percutaneous coronary intervention. Am J Cardiol 1995; 75:778-782

Angus DC, van der Poll T. Severe sepsis and septic shock. N Engl J Med 2013; 369(9):840-51.

Beinart R, Abu SR, Segev A et al. The incidence and clinical predictors of early stent thrombosis in patients with acute coronary syndrome. Am Heart J 2010; 159:118-124

Beinart SC, Kolm P, Veledar E et al. Long-term cost effectiveness of early and sustained dual oral antiplatelet therapy with clopidogrel given for up to one year after percutaneous coronary intervention results: from the Clopidogrel for the Reduction of Events During Observation (CREDO) trial. J Am Coll Cardiol 2005; 46:761-769

Boomer JS, To K, Chang KC et al. Immunosuppression in patients who die of sepsis and multiple organ failure. JAMA 2011; 306:2594-2605

Cauchy E, Cheguillaume B, Chetaile E. A controlled trial of prostacyclin and rt-tPA in the treatment of severe frostbite. NEJM 2011; 364:2

Charakida M, Donald AE, Terese M et al. Endothelial dysfunction in childhood infection. Circulation 2005; 111:1660-1665

Chen D, McVey JH, Dorling A. Enhanced effect of inhibition of thrombin on endothelium in murine endotoxaemia: Specific inhibition of thrombocytopenia. Thrombosis Res 2013; 132:750-756

Davies MG, Hagen PO. The vascular endothelium. A new horizon. Ann Surg 1993; 218:593-609
De Meyer GR, Martinet W. Autophagy in the cardiovascular system. Biochim Biophys Acta 2009; 1793:1485-1495

Fabbri LP, Nucera M, Al Malyan M et al. Regional anticoagulation and antiaggregation for CVVH in critically ill patients: a prospective, randomized, controlled pilot study. Acta Anaesthesiol Scand 2009

Fichtlscherer S, Breuer S, Zeiher AM. Prognostic value of systemic endothelial dysfunction in patients with acute coronary syndromes: further evidence for the existence of the "vulnerable" patient. Circulation 2004; 110:1926-1932

Fischell TA, Maheshwari A. Current applications for nicardipine in invasive and interventional cardiology. J Invasive Cardiol 2004; 16:428-432

Gatward JJ, Gibbon GJ, Wrathall G et al. Renal replacement therapy for acute renal failure: a survey of practice in adult intensive care units in the United Kingdom. Anaesthesia 2008; 63:959-966

Gimbrone MA, Jr., Aster RH, Cotran RS et al. Preservation of vascular integrity in organs perfused in vitro with a platelet-rich medium. Nature 1969; 222:33-36

Grande PO, Moller AD, Nordstrom CH et al. Low-dose prostacyclin in treatment of severe brain trauma evaluated with microdialysis and jugular bulb oxygen measurements. Acta Anaesthesiol Scand 2000; 44:886-894

Hansson GK. Inflammation, atherosclerosis, and coronary artery disease. N Engl J Med 2005; 352:1685-1695

Hingorani AD, Cross J, Kharbanda RK et al. Acute systemic inflammation impairs endothelium-dependent dilatation in humans. Circulation 2000; 102:994-999

Iwashyna TJ and Angus DC. Declining Case Fatality Rates for Severe Sepsis. Good Data Bring Good News With Ambiguous Implications. JAMA 2014; 311:1295-1297

Johansson PI, Ostrowski SR. Acute coagulopathy of trauma: balancing progressive catecholamine induced endothelial activation and damage by fluid phase anticoagulation. Med Hypotheses 2010; 75(6):564-7.

Kiernan TJ, Ruggiero NJ, Bernal JM et al. The no-reflow phenomenon in the coronary circulation. Cardiovasc Hematol Agents Med Chem 2009; 7:181-192

Kushner FG, Hand M, Smith SC, Jr. et al. 2009 focused updates: ACC/AHA guidelines for the management of patients with ST-elevation myocardial infarction (updating the 2004 guideline and 2007 focused update) and ACC/AHA/SCAI guidelines on percutaneous coronary intervention (updating the 2005 guideline and 2007 focused update) a report of the American College of Cardiology Foundation/American Heart Association Task Force on Practice Guidelines. J Am Coll Cardiol 2009; 54:2205-2241

Landmesser U, Drexler H. The clinical significance of endothelial dysfunction. Curr Opin Cardiol 2005; 20:547-551

Landmesser U, Hornig B, Drexler H. Endothelial function: a critical determinant in atherosclerosis? Circulation 2004; 109:II27-II33

Link A, Girnd M, Selejan S et al. Tirofiban preserves platelet loss during continuous renal replacement therapy in a randomised prospective open-blinded pilot study. Critical Care 2008; 12:R111

Mehta SR, Yusuf S, Peters RJ et al. Effects of pretreatment with clopidogrel and aspirin followed by long-term therapy in patients undergoing percutaneous coronary intervention: the PCI-CURE study. Lancet 2001; 358:527-533

Naredi S, Olivecrona M, Lindgren C et al. An outcome study of severe traumatic head injury using the "Lund therapy" with low-dose prostacyclin. Acta Anaesthesiol Scand 2001; 45:402-406

Patti G, Pasceri V, Melfi R et al. Impaired flow-mediated dilation and risk of restenosis in patients undergoing coronary stent implantation. Circulation 2005; 111:70-75

Peters RJ, Mehta SR, Fox KA et al. Effects of aspirin dose when used alone or in combination with clopidogrel in patients with acute coronary syndromes: observations from the Clopidogrel in Unstable angina to prevent Recurrent Events (CURE) study. Circulation 2003; 108:1682-1687

Shimokawa H, Yasuda S. Myocardial ischemia: Current concepts and future perspectives. J Cardiol 2008; 52:67-78

Solomon DH, Karlson EW, Rimm EB et al. Cardiovascular morbidity and mortality in women diagnosed with rheumatoid arthritis. Circulation 2003; 107:1303-1307

Uniform Requirements for Manuscripts Submitted to Biomedical Journals: Writing and Editing for Biomedical Publications. http://www.icmje.org/

Windeløv NA, Ostrowski SR, Perner A et al. Transfusion requirements and clinical outcome in intensive care patients receiving continuous renal replacement therapy: comparison of prostacyclin vs. heparin prefilter administration. BCF 2010; 21:414-419

Xiao Z, Theroux P, Frojmovic M. Modulation of platelet-neutrophil interaction with pharmacological inhibition of fibrinogen binding to platelet GPIIb/IIIa rector. Thromb Haemost 1999; 81:281-285

Appendix 1

26.marts 2013

PRODUKTRESUMÉ

for

Ilomedin, koncentrat til infusionsvæske, opløsning

0. D.SP.NR.
8177

1. LÆGEMIDLETS NAVN
ILOMEDIN

2. KVALITATIV OG KVANTITATIV SAMMENSÆTNING
1 ml vandig opløsning indeholder 27 mikrogram iloprost trometamol svarende til 20 mikrogram iloprost.

Hjælpestoffer, som behandleren skal være opmærksom på:
Natrium.
Ethanol.

Alle hjælpestoffer er anført under pkt. 6.1.

3. LÆGEMIDDELFORM
Koncentrat til infusionsvæske, opløsning.

Klar og uden partikler.

4. KLINISKE OPLYSNINGER

4.1 Terapeutiske indikationer
Behandling af fremskreden thromboangiitis obliterans (MB. Bürger) med kritisk ekstremitets-iskæmi i tilfælde, hvor revaskularisering ikke er indiceret.
Behandling af patienter med perifere kredsløbsforstyrrelser (PAOD), især ved risiko for amputation og hvor kirurgisk behandling eller angioplastik ikke er mulig.

4.2 Dosering og indgivelsesmåde
Bør kun anvendes under nøje kontrol på hospitaler eller ambulatorier, hvor der er tilstrækkeligt udstyr.

Graviditet bør udelukkes, før behandling af kvinder påbegyndes.

Ilomedin 20 mikrogram/ml administreres efter fortynding (se pkt. 6.6) som intravenøs infusion i en perifer vene eller i et centralt venøst kateter indenfor 6 timer daglig. Dosis skal justeres i forhold til patientens tolerans indenfor en dosis af 0,5 - 2,0 nanogram iloprost/kg/min.

Infusionsopløsningen skal fremstilles frisk hver dag for at sikre opløsningens sterilitet.
Indholdet af ampullen og fortyndingsvæsken skal blandes omhyggeligt.
Blodtryk og hjertefrekvens skal måles ved behandlingens start og efter hver forøgelse af dosis.

Den individuelt accepterede dosis fastsættes efter 2-3 dage. Til dette formål skal behandlingen starte med en infusionshastighed på 0,5 nanogram/kg/min. i 30 minutter. Med intervaller på 30 minutter skal dosis forøges i trin på 0,5 nanogram/kg/min. op til 2,0 nanogram/kg/min. Den nøjagtige infusionshastighed skal udregnes på basis af kropsvægten for at gennemføre en infusion inden for omfanget af 0,5 til 2,0 nanogram/kg/min. (se skema nedenfor ved anvendelse af infusionspumpe eller ved anvendelse af sprøjtepumpe).

Afhængig af forekomst af bivirkninger såsom hovedpine og kvalme eller af et uønsket blodtryksfald bør infusionshastigheden sænkes, indtil den accepterede dosis er fundet. Hvis bivirkningerne er alvorlige, bør infusionen stoppes. Behandlingen skal da fortsættes - normalt i 4 uger - med den dosis, der kan tolereres i de første 2-3 dage.

Der er to forskellige fortyndinger af en ampul, afhængig af infusionsteknikken. Den ene af de to fortyndinger har en 10 gange mindre koncentration end den anden (0,2 mikrogram/ml versus 2 mikrogram/ml) og må kun anvendes med en infusionspumpe (f.eks. Infusomat®). Den højere koncentrerede opløsning må kun anvendes med en sprøjtepumpe (f.eks. Perfusor®), se pkt 6.2.

- **Infusionshastigheder (ml/time) for forskellige doser ved anvendelse af infusionspumpe**

Den brugsklare infusionsopløsning kan indgives intravenøst ved hjælp af en infusionspumpe (f.eks. Infusomat®). Instruktioner vedrørende fortynding til brug med infusionspumpe se pkt. 6.6. Indholdet af ampullen og fortyndingsvæsken skal blandes omhyggeligt. I det tilfælde, hvor koncentrationen af Ilomedin er 0,2 mikrogram/ml, skal den anbefalede infusionshastighed bestemmes jvf. behandlingsskemaet nedenfor for at få en dosis indenfor området 0,5 til 2,0 nanogram/kg/min i relation til den enkelte patient.

(Aflæs venligst for at korrigere for patientens egentlige kropsvægt, sæt derefter infusionshastigheden til måldosis i nanogram/kg/min).

		Dosis (ng/kg/min)			
		0,5	1,0	1,5	2,0
		Infusionshastighed(ml/t)			
Legemsvægt (kg)	40	6,0	12	18,0	24
	50	7,5	15	22,5	30
	60	9,0	18	27,0	36
	70	10,5	21	31,5	42
	80	12,0	24	36,0	48
	90	13,5	27	40,5	54
	100	15,0	30	45,0	60
	110	16,5	33	49,5	66

- **Infusionshastigheder (ml/time) for forskellige doser ved anvendelse af sprøjtepumpe.**

En sprøjtepumpe med en 50 ml injektionssprøjte (f.eks. Perfusor®) kan også anvendes. Instruktioner vedrørende tilberedning til brug med sprøjtepumpe se pkt. 6.6. I det tilfælde, hvor koncentrationen af Ilomedin 20 mikrogram/ml er 2 mikrogram/ml, skal den anbefalede infusionshastighed bestemmes jvf. behandlingsskemaet nedenfor for at få en dosis inden for området 0,5 til 2,0 nanogram/kg/min i relation til den enkelte patients vægt (se skema nedenfor til anvendelse med sprøjtepumpe).

Aflæs venligst for at korrigere for patientens egentlige legemsvægt, sæt derefter infusionshastigheden til måldosis i nanogram/kg/min.

		Dosis (ng/kg/min)			
		0,5	1,0	1,5	2,0
		Infusionshastighed(ml/t)			
	40	0,60	1,2	1,80	2,4
	50	0,75	1,5	2,25	3,0
Legemsvægt	60	0,90	1,8	2,70	3,6
(kg)	70	1,05	2,1	3,15	4,2
	80	1,20	2,4	3,60	4,8
	90	1,35	2,7	4,05	5,4
	100	1,50	3,0	4,50	6,0
	110	1,65	3,3	4,95	6,6

En behandling varer op til 4 uger.

Kontinuert infusion over adskillige dage kan ikke anbefales, da det er muligt, at der udvikles tachyphylaxi af blodpladerne og risiko for, at blodpladerne genbindes med hyperaggregation ved behandlingens ophør. Denne forholdsregel tages til trods for, at der ikke har været rapporteret kliniske komplikationer af denne art.

Nedsat nyre- eller leverfunktion
Det bør tages i betragtning, at hos patienter med nyresvigt, som kræver dialyse, og hos patienter med levercirrhose er eliminationen af iloprost reduceret. Hos disse patienter er en dosisreduktion nødvendig (f.eks. det halve af den anbefalede dosis).

Børn og unge
Erfaring med behandling af børn er begrænset.

4.3 Kontraindikationer

Ilomedin er kontraindiceret i følgende tilfælde:

- Overfølsomhed over for iloprost eller over for et eller flere af hjælpestofferne anført i pkt. 6.1.
- Graviditet (se pkt. 4.6).
- Alvorlige koronar hjertesygdom eller ustabil angina.
- Myokardieinfarkt indenfor de sidste 6 måneder.
- Akut eller kronisk kongestiv hjerteinsufficiens (NYHA II-IV).
- Ikke kontrollerbare arytmier eller formodet lungestase.
- Situationer hvor det kan forventes, at virkningen af Ilomedin på trombocytterne giver øget risiko for blødning (f.eks. aktivt peptic ulcus, traumer, interkraniel blødning).

4.4 Særlige advarsler og forsigtighedsregler vedrørende brugen

Operation bør ikke forsinkes hos patienter, der kræver akut amputation, f.eks. ved inficeret gangræn.

Patienten bør stærkt frarådes al rygning.

Eliminationen af iloprost er nedsat hos patienter med nedsat leverfunktion og hos patienter med nedsat nyrefunktion, som kræver dialyse (se pkt. 4.2 og 5.2).

Hos patienter med lavt blodtryk skal man være opmærksom for at undgå yderligere hypotension og patienter med en alvorlig hjertesygdom bør overvåges nøje.

Der skal tages hensyn til muligheden for ortostatisk hypotension hos patienter, der rejser sig fra liggende til opret stilling efter endt behandling.

Hos patienter med et cerebrovaskulært tilfælde (f.eks. forbigående iskæmisk tilfælde (TSI), stroke) indenfor de seneste 3 måneder skal der foretages en omhyggelig risikovurdering (se også pkt. 4.3: blødningsrisiko, f.eks. intrakraniel blødning).

Særlige forsigtighedsregler
Paravaskulær infusion af ufortyndet Ilomedin 20 mikrogram/ml kan føre til lokale forandringer ved injektionsstedet.
Peroral indtagelse og kontakt med slimhinderne bør undgås.

Ved kontakt med huden kan iloprost bevirke længerevarende, men smertefri erytem. Passende forholdsregler skal derfor tages for at forhindre kontakt mellem iloprost og huden. Hvis iloprost kommer i kontakt med huden, skal det pågældende område omgående vaskes med rimelige mængder vand eller saltvand.

Hjælpestoffer
Dette lægemidlet indeholder en mindre mængde ethanol, mindre end 100 mg pr. dosis.

Dette lægemidlet indeholder mindre end 1 mmol natrium pr. dosis, dvs. stort set "natriumfri".

4.5 Interaktion med andre lægemidler og andre former for interaktion
Farmakologiske undersøgelser har vist, at iloprost har additiv virkning på den antihypertensive aktivitet af beta-receptorblokkere, calciumantagonister og vasodilatorer, og en potenserende effekt på ACE-hæmmeres antihypertensive virkning. Hvis en signifikant hypotension opstår, kan denne korrigeres ved en reduktion af iloprost-dosis.

Da iloprost hæmmer blodpladernes funktion kan det øge risikoen for blødning, når det anvendes sammen med heparin eller antikoagulantia af coumarin-typen, eller hæmmere af trombocytaggregation f.eks. acetylsalicylsyre, NSAID-præparater og fosfodiesterase-hæmmere. Hvis en sådan situation opstår, skal iloprost-infusionen stoppes. Peroral præmedicinering med acetylsalicylsyre op til 300 mg i en periode på 8 dage havde ingen effekt på iloprosts farmakokinetik. Forsøg hos mennesker viser, at infusioner med iloprost ikke påvirker farmakokinetikken af gentagne perorale digoxindoser og at iloprost ikke har nogen effekt på farmakokinetikken af samtidig administreret t-PA.

På trods af at der ikke er udført kliniske studier, viste *in vitro* studier, hvori det inhibitoriske potentiale af iloprost på aktiviteten af cytochrom P450 enzymer blev undersøgt, at der ikke kan forventes nogen betydende hæmning af stoffets metabolisme via disse enzymer med iloprost.

Ilomedin, koncentrat til infusionsvæske, opløsning indeholder ethanol, se pkt. 4.4.

4.6 Graviditet og amning
Fertilitet
Der er ikke set virkninger på fertiliteten hos han- og hunrotter i korrekt gennemførte ikke-kliniske studier med rotter.

Graviditet
Ilomedin er kontraindiceret (se pkt. 4.3) under graviditet.
Data for anvendelse af iloprost til gravide er utilstrækkeligt. Reproduktionstoksicitet er set hos rotter, men ikke hos kaniner og aber (se pkt. 5.3). Den potentielle risiko for mennesker kendes ikke.

Kvinder i den fertile alder skal anvende sikker antikontraception under behandlingen.

Amning

Det vides ikke om iloprost udskilles i modermælken hos mennesker. Dyreforsøg har vist, at meget små mængder af iloprost udskilles i rottemælk (se sektion 5.3). En risiko for det ammende barn kan ikke udelukkes og amning bør undgås under behandling med Ilomedin.

4.7 Virkning på evnen til at føre motorkøretøj eller betjene maskiner
Ikke mærkning.
Ilomedin kan især i starten af behandlingen og ved øgning af dosis påvirke evnen til at føre motorkøretøj eller betjene maskiner i mindre eller moderat grad.

4.8 Bivirkninger
Oversigt over sikkerhedsprofilen
Sikkerhedsprofilen for Ilomedin er baseret på oplysninger fra overvågning efter markedsføringen og på poolede oplysninger fra kliniske studier. De ujusterede incidenser er baseret på den kumulative database på 3325 patienter, der har fået iloprost enten i kontrollerede eller i ikke-kontrollerede kliniske studier eller i et program med særlig udleveringstilladelse. Patienterne er generelt ældre og/eller multimorbide patienter med fremskreden perifer atherosclerotisk karsygdom (sv.t. Fontaine stadie III og IV) samt patienter med thromboangiitis obliterans, se detaljer i tabel 1.

De hyppigst sete bivirkninger (≥ 10%) hos patienter, der fik iloprost i kliniske studier, var hovedpine, flushing, kvalme, opkastning og hyperhidrose. Disse bivirkninger er sandsynlige medens dosis titreres ved behandlingens start for at finde den dosis, der bedst tolereres af hver patient. Disse bivirkninger forsvinder dog normalt hurtigt, når dosis reduceres.

De mest alvorlige bivirkninger hos patienter, der får iloprost, er: cerebrovaskulær lidelse, myokardieinfarkt, lungeemboli, hjertesvigt, krampe, hypotension, takykardi, astma, angina pectoris, dyspnø og lungeødem.

En anden gruppe bivirkninger vedrører lokale reaktioner ved infusionsstedet. F.eks. kan der opstå rødmen og smerte ved infusionsstedet, eller en kutan vasodilation kan medføre stribeformet erytem over infusionsvenen.

Skematisk oversigt over bivirkninger
De bivirkninger, der er set med ilomedin, er vist i tabellen nedenfor. De er klassificeret i henhold til systemorganklasser (MedDRA version 14.1). Der er anvendt den mest passende MedDRA-betegnelse til at beskrive en bestemt reaktion og dens synonymer og tilhørende tilstande.

Bivirkninger fra kliniske studier er klassificeret i henhold til deres hyppigheder. Hyppighedsgrupperne er defineret på følgende måde: Meget almindelig ≥1/10, almindelig ≥1/100 til <1/10, ikke almindelig ≥1/1.000 til <1/100 og sjælden ≥1/10.000 til <1/1.000.

Tabel 1: Bivirkninger, der er set i kliniske studier eller under overvågningen efter markedsføringen hos patienter, der bliver behandlet med Ilomedin

Systemorganklasse (MedDRA)	Meget almindelig	Almindelig	Ikke almindelig	Sjælden
**Blod og lymfesystem**			Trombocytopeni	
**Immunsystemet**			Hypersensitivitet	
**Metabolisme og ernæring**		Nedsat appetit		
**Psykiske forstyrelser**		Apati, konfusion	Angst, depression, hallucinationer	

**Systemorganklasse (MedDRA)**	**Meget almindelig**	**Almindelig**	**Ikke almindelig**	**Sjælden**
**Nervesystemet**	Hovedpine	Svimmelhed/vertigo, paræstesi/prikkende, snurrende fornemmelse, Hyperæstesi/brænden de fornemmelse, rastløs uro/agitation, sedering, døsighed	Konvulsioner*, synkope, tremor, migræne	
**Øjne**			Sløret syn, øjenirritation, øjensmerter	
**Øre og labyrint**				Vestibulær sygdom
**Hjerte**		Takykardia*, bradykardi, angina pectoris*	Myokardieinfarkt*, hjertesvigt*, arytmi/ ekstrasystoler	
**Vaskulære sygdomme**	Flushing	Hypotension*, forhøjet blodtryk	Cerebrovasculær lidelse* / cerebral iskæmi, lungeimboli*, dyb venetrombose	
**Luftveje, thorax og mediastinum**		Dyspnø*	Astma*, lungeødem*	Hoste
**Mave-tarm-kanalen**	Kvalme, opkastning	Diaré, mavebesvær /mavesmerter	Hæmoragisk diaré, rektalblødning, dyspepsi, rektal tenesmus, forstoppelse, opstød, dysfagi, tør mund/smagsforstyrr elser	Proctitis
**Lever og galdeveje**			Gulsot	
**Hud og subkutane væv**	Hyperhidrose		Pruritus	
**Knogler, led, muskler og bindevæv**		Kæbesmerte, trismus, myalgi, artralgi	Tetani, muskelkramper, hypertoni	
**Nyrer og urinveje**			Nyresmerter, tenesmus vesicae, urinabnormitet, dysuri,	

Systemorganklasse (MedDRA)	Meget almindelig	Almindelig	Ikke almindelig	Sjælden
			urinvejsforstyrrelser	
**Almene symptomer og reaktioner på administrationssted et**		Smerte, pyreksi/øget legemstemperatur, generel varmefornemmelse, asteni, utilpashed, kulderystelser, træthed, tørst, reaktioner ved infusionsstedet (erytem, smerte, flebit på infusionsstedet).		

* livstruende tilfælde og/eller dødsfald er set.

Iloprost kan udløse angina pectoris, især hos patienter med koronararteriesygdom.

Risikoen for blødning er øget hos patienter, når der samtidig gives trombocytaggregationshæmmere, heparin eller antikoagulantia af coumarintypen.

4.9 Overdosering

Symptomer
Hypotensiv reaktion kan forventes ligesom ansigtsødem, hovedpine, svedudbrud, kvalme, opkastning, krampelignende mavesmerter og diaré. Øget blodtryk, bradykardi eller takykardi samt smerte i ekstremiteter eller ryg kan forekomme.

Behandling
Afbrydelse af iloprostinfusionen, overvågning og symptomatiske forholdsregler.
En antidot er ikke kendt.

4.10 Udlevering

B

5. FARMAKOLOGISKE EGENSKABER

5.0 Terapeutisk klassifikation

B 01 AC 11 – Antithrombosemidler, blodplade-aggregationshæmmere excl. heparin.

5.1 Farmakodynamiske egenskaber

Iloprost er en syntetisk prostacyklinanalog. Følgende farmakologiske virkninger er påvist:

Hæmning af blodpladeaggregation, blodpladeadhæsion og frigørelsesreaktion, dilatation af arteriolerne og venolerne, øgning af den kapilære densitet og reduktion af den øgede vasku-lære permeabilitet i mikrocirkulationen forårsaget af f.eks. sertonin eller histamin, stimulering af endogent fibrinolytisk potentiale, hæmning af leukocytadhæsion efter læsion af endotelet og af leukocytakkumulering i læderet væv og nedsat frigivelse af tumornekrosefaktor.

5.2 Farmakokinetiske egenskaber

- Distribution

Steady state plasmakoncentrationer opnås så hurtigt som 10-20 minutter efter starten af den intravenøse infusion. Steady state plasmakoncentrationer er lineært forbundet til infusions-hastigheden. Plasmakoncentrationer på omkring 135 +/- 24 pg/ml opnås ved en infusions-hastighed på 3 ng/kg/min. Plasmakoncentrationen af iloprost falder meget hurtigt efter endt infusion pga. den hurtige metabolisering. Den metaboliske clearance af stoffet fra plasma er ca. 20 +/- 5 ml/kg/min. Halveringstiden i eliminationsfasen fra plasma er 0,5 timer. Et resultat heraf er, at stoffets koncentration falder til mindre end 10 % af ligevægtskoncentrationen 2 timer efter endt infusion. Iloprosts farmakokinetik er uafhængig af patientens alder og køn. Imidlertid er Iloprost clearance reduceret med en faktor 2-4 hos patienter med levercirrhose og hos patienter med kronisk nyresvigt, som kræver dialyse.

Interaktioner med andre lægemidler på plasmaproteinbindingsniveau er usandsynlig, fordi den største del af iloprost er bundet til albumin i blodplasma (60 % proteinbinding) og kun meget små koncentrationer iloprost opnås. En påvirkning af iloprost behandling på biotrans-formationen af andre lægemidler er ligeledes usandsynlig pga. iloprosts metabolisme og den ekstremt lave dosis.

- Metabolisme

Iloprost metaboliseres hovedsagelig via en β-oxidation af carboxyl-sidekæden. Intet uomdan-net stof elimineres. Hovedmetabolitten er tetranor-iloprost, som findes i urinen i en fri og i en konjugeret form i 4 diastereoisomere. Tetranor-iloprost er farmakologisk inaktiv som vist i dyrestudier. *In vitro*-studier antyder, at metabolismen af iloprost i lungerne er den samme efter intravenøs administration eller inhalation.

- Elimination

Hos forsøgspersoner med normal nyre- eller leverfunktion er tilgængeligheden af iloprost efter intravenøs infusion i de fleste tilfælde karakteriseret med en to-fase profil med gennem-snitlige halveringstider på 3-5 min og 15 til 30 min. Den totale clearance af iloprost er ca. 20 ml/kg/min, hvilket tyder på ekstrahepatisk medvirken ved iloprosts metabolisme.

I et masse/balance studie hos raske forsøgspersoner med brug af ^{3}H-iloproster genfindingen efter intravenøs infusion af total radioaktivitet 81 % og genfindingen i urin og fæces er hen-holdsvis 68 % og 12 %. Metabolitterne elimineres fra plasma og med urinen i 2 faser, for hvilke der er halveringstider på 2 og 5 timer (plasma) og for 2 -18 timer (urinen).

- Karakteristika hos patienter

Nedsat nyrefunktion

I et studie med intravenøs infusion af iloprost er det vist, at patienter i slutstadiet af nyreinsuf-ficiens i intermitterende dialysebehandling har en signifikant lavere clearance (gennemsnit clearance = 5±2 ml/min/kg) end den, der er set hos patienter med nyreinsufficiens, som ikke får intermitterende dialysebehandling (gennemsnitlig clearance = 18±2 ml/min/kg).

Hepatisk dysfunktion

Plasmakoncentrationerne af iloprost er påvirket af ændringer i leverfunktionen, eftersom det hovedsaglig metaboliseres i leveren. I et intravenøst studie af 8 patienter med levercirrhose var de opnåede resultater, at den gennemsnitlige clearance af iloprost blev estimeret til 10 ml/min/kg.

Alder og køn

Alder og køn er ikke af klinisk relevans for iloprosts farmakokinetik.

5.3 Prækliniske sikkerhedsdata

De ikke-kliniske data viste ingen speciel fare for mennesker på basis af traditionelle studier af sikkerhedsfarmakologi, gentagen dosistoksicitet, genotoksicitet, karcinogenicitet. Prækliniske effekter fremkom kun ved en eksponering, der blev anset for i en sådan grad at overstige den maksimale eksponering for mennesker, at det næppe er klinisk relevant.

- Systemisk toksicitet

Resultater fra undersøgelser for akut toksicitet tyder ikke på nogen risiko for akutte bivirkninger hos mennesker, når det høje farmakologiske potentiale af iloprost og den absolutte dosis, der er nødvendig til terapeutisk anvendelse, blev taget i betragtning. Som forventet for et prostacyklin, så giver iloprost hæmodynamiske virkninger (vasodilation, rødme af huden, hypotension, hæmning af blodpladefunktionen, åndedrætsbesvær) og almindelige tegn på forgiftning, såsom apati, smagsforandringer og posturale forandringer.

I systemiske toksicitetsundersøgelser med gentagen (kontinuert) i.v infusion forekom en let reduktion i blodtrykket ved doser over 14 ng/min. og alvorlige uønskede virkninger (hypotension, åndedrætsforstyrrelser) forekom kun efter ekstremt høje doser.

- Genotoksisk potentiale

In-vitro og *in-vivo* studier for genotoksisk effekt har ikke frembragt bevis for et mutagent potentiale.

- Reproduktionstoksikologi

Ved embryo- og foetus-toksicitetsstudier i rotter medførte kontinuerlig intravenøs behandling med iloprost misdannelse af enkelte falanks i forpoterne hos nogle få fostre/unger uafhængigt af dosis. Disse ændringer regnes ikke for virkelig teratogene virkninger, men skyldes sand-synligvis iloprost-induceret væksthæmning i den sene organogenese på grund af hæmodyna-miske ændringer i pars fetalis placentae. Det må formodes, at denne væksthæmning i stor udstrækning er reversibel under den postnatale udvikling. Der blev ikke observeret sådanne misdannelser eller andre strukturelle abnormiteter i lignende embryotoksicitetsstudier med kaniner og aber, selv ved betydeligt højere doser, der er mange gange større end dosis til mennesker.

Meget små mængder af ilomedin og/eller ilomedin metabolitter blev udskilt i rottemælk (under 1% af den intravenøse iloprost dosis).

6. FARMACEUTISKE OPLYSNINGER

6.1 Hjælpestoffer

Trometamol, ethanol 96 % v/v, natriumchlorid, saltsyre, 1N, sterilt vand.

6.2 Uforligeligheder

Da der er risiko for interaktioner, må intet andet lægemiddel tilføres den brugsklare opløsning.

6.3 Opbevaringstid

5 år.

6.4 Særlige opbevaringsforhold

Ingen særlige opbevaringsbetingelser.

6.5 Emballagetyper og pakningsstørrelser

Ampuller på 1 ml og 3 ml, glastype I, farveløs.
1 ampul indeholder 1 ml eller 2,5 ml koncentrat til infusionsvæske, opløsning.
5 ampuller indeholder hver 1 ml eller 2,5 ml koncentrat til infusionsvæske, opløsning.

Ikke alle pakningsstørrelser er nødvendigvis markedsført.

6.6 Regler for destruktion og anden håndtering

Må kun bruges efter fortynding.

Den brugsklare infusionsopløsning skal fremstilles frisk hver dag for at sikre opløsningens sterilitet.

Instruktion vedrørende fortynding

Indholdet af ampullen og fortyndingsvæsken skal blandes omhyggeligt.

Fortynding af Ilomedin til brug med infusionspumpe:

Til dette brug skal indholdet af en 2,5 ml ampul (dvs. 50 µg) af Ilomedin 20 mikrogram/ml fortyndes med steril, fysiologisk saltopløsning eller en 5 % glucoseopløsning op til 250 ml, og indholdet af en 1 ml ampul (dvs. 20 µg) af Ilomedin 20 mikrogram/ml skal fortyndes med steril, fysiologisk saltopløsning eller en 5 % glukoseopløsning op til 100 ml.

Fortynding af Ilomedin til brug med sprøjtepumpe:

I dette tilfælde skal indholdet af en 2,5 ml ampul (dvs. 50 µg) af Ilomedin 20 mikrogram/ml fortyndes med steril, fysiologisk saltopløsning eller 5 % glukoseopløsning for at nå en endelig mængde på 25 ml, og indholdet af en 1 ml ampul (dvs. 20 µg) af Ilomedin 20 mikrogram/ml bliver fortyndet med steril, fysiologisk saltopløsning eller 5 % glukoseopløsning for at nå en endelig mængde på 10 ml.

Ikke anvendt lægemiddel samt affald heraf skal bortskaffes i henhold til lokale retningslinjer.

7. INDEHAVER AF MARKEDSFØRINGSTILLADELSEN

Bayer Pharma AG
D-13342 Berlin
Tyskland

Repræsentant

Bayer A/S
Arne Jacobsens Allé 13
2300 København S

8. MARKEDSFØRINGSTILLADELSESNUMMER

19398

9. DATO FOR FØRSTE MARKEDSFØRINGSTILLADELSE

6. februar 1992

10. DATO FOR ÆNDRING AF TEKSTEN

26. marts 2013

Appendix 2

LÆGEMIDDEL STYRELSEN

PRODUKTRESUME

1. LÆGEMIDLETS NAVN

INTEGRILIN 2 mg/ml injektionsvæske, opløsning

2. KVALITATIV OG KVANTITATIV SAMMENSÆTNING

Hver ml opløsning indeholder 2 mg/ml eptifibatid.

Et hætteglas med 10 ml opløsning indeholder 20 mg eptifibatid.

Alle hjælpestoffer er anført under pkt. 6.1.

3. LÆGEMIDDELFORM

Injektionsvæske, opløsning
Klar, farveløs opløsning

4. KLINISKE OPLYSNINGER

4.1 Terapeutiske indikationer

INTEGRILIN er beregnet til anvendelse sammen med acetylsalicylsyre og ufraktioneret heparin.

INTEGRILIN er indiceret til forebyggelse af tidligt opstående myokardieinfarkt hos voksne med ustabil angina eller non-Q-tak-myokardieinfarkt med det sidste anfald af brystsmerter inden for 24 timer og med elektrokardiogram (ekg)-ændringer og/eller forhøjede hjerteenzymer.

De patienter, som mest sandsynligt vil have gavn af INTEGRILIN behandling, er de, som har høj risiko for at udvikle myokardieinfarkt inden for de første 3–4 dage, efter at de akutte anginasymptomer debuterede inklusive fx de patienter, som med stor sandsynlighed skal have foretaget en tidlig PTCA (perkutan transluminal koronar angioplastik) (se pkt. 5.1).

4.2 Dosering og indgivelsesmåde

Dette produkt er udelukkende beregnet til hospitalsbrug. Produktet bør administreres af speciallæger med erfaring i behandling af akutte koronarsyndromer.

INTEGRILIN infusionsvæske, opløsning skal anvendes i forbindelse med INTEGRILIN injektionsvæske, opløsning.

Samtidig administration af heparin anbefales, medmindre det er kontraindiceret, som ved anamnese med trombocytopeni i forbindelse med anvendelse af heparin (se ”Heparin-indgift” pkt. 4.4). INTEGRILIN er også

beregnet til anvendelse samtidig med acetylsalicylsyre, idet dette er en del af standardbehandlingen af patienter med akutte koronarsyndromer, medmindre anvendelse er kontraindiceret.

Voksne (≥ 18 år) med ustabil angina (UA) eller non-Q-tak-myokardieinfarkt (NQMI)
Den anbefalede dosering er intravenøs bolus på 180 mikrogram/kg indgivet snarest muligt, efter diagnosen er stillet, efterfulgt af kontinuerlig infusion af 2,0 mikrogram/kg/minut i op til 72 timer indtil påbegyndelse af koronararterie bypass-kirurgi (CABG) eller indtil udskrivning fra hospitalet (hvad, der forekommer først). Hvis der foretages perkutan koronar-intervention (PCI) under behandling med eptifibatid, skal infusionen fortsættes i 20-24 timer efter PCI med en samlet maksimal behandlingsvarighed på 96 timer.

Akut eller semi-elektiv kirurgi
Hvis patienten har behov for akut eller øjeblikkelig hjertekirurgi under behandlingen med eptifibatid, så afslut straks infusionen. Hvis patienten behøver semi-elektiv kirurgi, så stop infusionen med eptifibatid på et passende tidspunkt for at give tid til, at trombocytfunktionen kan vende tilbage til den normale.

Leverinsufficiens
Erfaringer med patienter med leverinsufficiens er meget begrænsede. Indgives forsigtigt til patienter med leverinsufficiens, hos hvem koagulationen kan være påvirket (se pkt. 4.3, protrombintid). Kontraindiceret hos patienter med klinisk signifikant leverinsufficiens.

Nyreinsufficiens
Hos patienter med moderat nedsat nyrefunktion (kreatininclearance ≥ 30-< 50 ml/minut) skal der gives en intravenøs bolusinjektion på 180 mikrogram/kg efterfulgt af 1 mikrogram/kg/minut i kontinuerlig infusion i resten af behandlingsperioden. Anvendelse hos patienter med mere alvorlig nyreinsufficiens er kontraindiceret (se pkt. 4.3).

Pædiatrisk population
Anbefales ikke til børn og unge under 18 år på grund af utilstrækkelige oplysninger om sikkerhed og effekt.

4.3 Kontraindikationer

INTEGRILIN må ikke anvendes til behandling af patienter med:
- overfølsomhed over for det aktive stof eller over for et eller flere af hjælpestofferne
- tegn på gastrointestinal blødning, kraftig genital/urinal blødning eller anden aktiv abnorm blødning inden for de sidste 30 dage før behandling

anamnese med apopleksi inden for 30 dage eller en hvilket som helst tidligere hjerneblødning
tidligere kendt sygdom inden for kraniet (neoplasi, arterio-venøs misdannelse, aneurisme)
- større operation eller alvorligt traume inden for de seneste 6 uger
- anamnese med blødningstendens
- trombocytopeni (< 100.000 celler/mm^3)
- protrombintid > 1,2 gange kontrol eller International Normaliseret Ratio (INR) ≥ 2,0
- svær hypertension (systolisk blodtryk > 200 mm Hg eller diastolisk blodtryk > 110 mm Hg under antihypertensiv behandling)
- svær nyreinsufficiens (kreatininclearance < 30 ml/minut) eller dialysepatienter
- klinisk signifikant nedsat leverfunktion
- samtidig eller planlagt indgift af en anden parenteral glykoprotein-(GP) IIb/IIIa-hæmmer

4.4 Særlige advarsler og forsigtighedsregler vedrørende brugen

Blødning
INTEGRILIN er et antitrombotisk stof, der virker ved at hæmme trombocytaggregationen og patienten skal derfor observeres omhyggeligt for tegn på blødning under behandling (se pkt. 4.8). Kvinder, ældre, patienter med lav kropsvægt eller med moderat nyreinsufficiens (kreatininclearance ≥30 - <50 ml/min) kan have en øget blødningsrisiko. Overvåg disse patienter omhyggeligt for blødninger.

Blødning er mest almindeligt ved det arterielle indstikssted hos patienter, der gennemgår perkutan arteriel intervention. Alle potentielle blødningssteder, såsom kateterindføringssteder, arterielle, venøse eller kanyle-injektionssteder, operationssteder, gastrointestinale og genitourinare veje, skal observeres omhyggeligt. Andre mulige blødningssteder, såsom det centrale og perifere nervesystem og retroperitoneale steder, skal også kontrolleres nøje.

Da INTEGRILIN hæmmer trombocytaggregationen, skal der udvises forsigtighed, når det anvendes med andre stoffer, der påvirker hæmostasen, inklusive ticlopidin, clopidogrel, trombolytika, orale antikoagulantia, dextranopløsninger, adenosin, sulfinpyrazon, prostacyclin, non-steroide antiinflammatoriske stoffer, eller dipyramidol (se pkt. 4.5).

Der er ingen erfaringer med INTEGRILIN og lavmolekylære hepariner.

Der er begrænsede terapeutiske erfaringer med INTEGRILIN til patienter med generel indikation for trombolytisk behandling (f.eks. akut transmural myokardieinfarkt med nye patologiske Q-takker eller forhøjet ST-segmenter eller venstresidigt ledningsblok i ekg). INTEGRILIN bør derfor ikke anvendes i sådanne tilfælde (se pkt. 4.5).

Infusion med INTEGRILIN skal afbrydes øjeblikkeligt, hvis der opstår situationer, der kræver trombolytisk behandling, eller hvis patienten skal gennemgå en akut CABG-operation eller har behov for en ballonpumpe i aorta.

Hvis der forekommer alvorlig blødning, der ikke kan kontrolleres med tryk, skal infusionen med INTEGRILIN og eventuel ufraktioneret heparin, der gives samtidig, stoppes øjeblikkeligt.

Arterielle procedurer
Under behandling med eptifibatid er der en betydelig stigning i blødningshyppigheden, især i det femorale arterieområde, hvor kateterspidsen indføres. Vær omhyggelig med at sikre, at der kun stikkes gennem den forreste væg af den femorale arterie. Arteriespidsen kan fjernes, når koagulationen er vendt tilbage til det normale, svarende til at aktiveret koagulationstid (ACT) er mindre end 180 sekunder (almindeligvis 2-6 timer efter seponering af heparin). Efter fjernelse af indføringsspidsen skal omhyggelig hæmostase sikres under nøje observation.

Trombocytopeni
INTEGRILIN hæmmer trombocytaggregationen, men synes ikke at påvirke trombocytternes levedygtighed. Som vist i kliniske undersøgelser var forekomsten af trombocytopeni lav og på samme niveau hos de patienter, der fik behandling med eptifibatid og dem, der fik placebo. Ved administration af eptifibatid er der observeret trombocytopeni, samt akut kraftig trombocytopeni (se pkt. 4.8). Trombocyttællinger bør overvåges i følgende tilfælde: før behandling, inden for 6 timer efter administration, og efterfølgende mindst én gang daglig under behandling og straks i tilfælde af kliniske symptomer på uventet blødningstendens. Hvis patienten får et betydeligt fald i trombocytterne til < 100.000/mm^3, så afbryd INTEGRILIN og ufraktioneret heparin samt kontrollér patienten på passende måde. En beslutning om at foretage blodpladetransfusion bør baseres på individuel klinisk vurdering. Der er ingen data med anvendelse af INTEGRILIN til patienter med tidligere trombocytopeni efter andre parenterale GP IIb/IIIa-hæmmere, og disse patienter kræver således nøje overvågning, som ovenfor beskrevet.

Heparin-indgift
Heparin-indgift anbefales, medmindre der er en kontraindikation for dette (som ved anamnese med trombocytopeni i forbindelse med brug af heparin).

UA/NQMI: Til en patient, der vejer ≥ 70 kg, anbefales det at give en bolusdosis på 5.000 enheder efterfulgt af en konstant intravenøs infusion på 1.000 enheder/time. Hvis patienten vejer < 70 kg, anbefales en bolusdosis på 60 enheder/kg, efterfulgt af en infusion på 12 enheder/kg/time. Den aktiverede, partielle tromboplastintid (aPTT) skal følges for at bibeholde en værdi mellem 50 og 70 sekunder. Over 70 sekunder kan der være øget risiko for blødning.

Hvis der skal foretages PCI med udførelse som for UA/NQMI, så hold øje med den aktiverede koagulationstid (ACT) for at bibeholde en værdi mellem 300-350 sekunder. Seponer heparin-indgift, hvis ACT overstiger 300 sekunder. Indgiv ikke heparin, før ACT falder til under 300 sekunder.

Overvågning af laboratorieværdier
Før infusion med INTEGRILIN anbefales det, at man foretager følgende laboratorieundersøgelser for at identificere præeksisterende hæmostatiske abnormaliteter: Protrombintid (PT) og aPTT, serumkreatinin, trombocyttal, hæmoglobin og hæmatokritværdier. Hæmoglobin, hæmatokrit og blodpladetal skal kontrolleres både inden for 6 timer efter behandlingsstart og mindst én gang daglig, derefter under behandling (eller oftere, hvis der er tegn på et mærkbart fald). Hvis blodpladetallet falder til < 100.000/mm^3, er yderligere trombocyttællinger påkrævet for at udelukke pseudotrombocytopeni. Seponer ufraktioneret heparin. Mål også ACT hos patienter, der gennemgår PCI.

Immunogenicitet
Immunogenisk respons eller antistoffer mod eptifibatid er set i isolerede tilfælde hos naive patienter eller i sjældne tilfælde hos patienter efter reeksponering af eptifibatid. Der er kun begrænsede erfaringer med gentagen anvendelse af INTEGRILIN. Ved gentagen behandling med INTEGRILIN er der derfor ingen forventning om formindsket terapeutisk respons.

4.5 Interaktion med andre lægemidler og andre former for interaktion

Warfarin og dipyridamol
INTEGRILIN synes ikke at give en øget risiko for større eller mindre blødninger i forbindelse med samtidig brug af warfarin og dipyridamol. INTEGRILIN-behandlede patienter, der havde en protrombintid (PT) > 14,5 sekunder og fik warfarin samtidig, syntes ikke at have øget risiko for blødning.

INTEGRILIN og trombolytiske stoffer
Data er begrænsede for anvendelse af INTEGRILIN til patienter, der får trombolytiske stoffer. Hverken i et PCI-studie eller i et akut myokardieinfarkt-studie observeredes der konsistente tegn på, at eptifibatid øger risikoen for større og mindre blødninger associeret til vævsplasminogen-aktivator. I et akut myokardieinfarkt-studie syntes eptifibatid at øge risikoen for blødning ved indgift med streptokinase. Kombination af reduceret dosis af tenecteplase og eptifibatid sammenlignet med placebo og eptifibatid øgede signifikant risikoen for både større og mindre blødninger, da de blev administreret samtidig i et akut ST-elevations myokardieinfarkt-studie.

I et akut myokardieinfarkt-studie med 181 patienter blev eptifibatid givet (i regimer med bolusinjektion på op til 180 mikrogram/kg, efterfulgt af infusion på op til 2 mikrogram/kg/minut i op til 72 timer) sammen med streptokinase (1,5 millioner enheder over 60 minutter). Ved de højeste undersøgte infusionshastigheder (1,3 mikrogram/kg/minut og 2,0 mikrogram/kg/minut) var eptifibatid forbundet med en øget hyppighed af blødning og transfusioner sammenlignet med den forekomst, der sås, når streptokinase blev givet alene.

4.6 Graviditet og amning

Der foreligger ikke tilstrækkelige data om brugen af eptifibatid hos gravide kvinder.
De udførte dyreforsøg er utilstrækkelige med hensyn til virkningerne for graviditetens, embryoets/fostrets udvikling, fødslen eller den postnatale udvikling (se pkt. 5.3). Den potentielle risiko for mennesker er ukendt. INTEGRILIN bør ikke anvendes under graviditet, medmindre det er klart nødvendigt.

Det vides ikke, om eptifibatid udskilles i modermælk. Det anbefales at afbryde amning under behandlingsperioden.

4.7 Virkning på evnen til at føre motorkøretøj eller betjene maskiner

Ikke relevant, da INTEGRILIN kun er beregnet til patienter indlagt på hospital.

4.8 Bivirkninger

De fleste bivirkninger hos de patienter, der fik behandling med eptifibatid, var generelt relateret til blødninger eller til kardiovaskulære hændelser, der forekommer hyppigt hos denne patientpopulation.

Kliniske undersøgelser

Data kilder anvendt til at bestemme hyppighed af bivirkninger inkluderede to kliniske fase III undersøgelser (PURSUIT og ESPRIT). Disse undersøgelser er beskrevet kort nedenfor.

PURSUIT: Dette var en randomiseret, dobbeltblindet vurdering af virkning og sikkerhed af Integrilin *versus* placebo til at reducere mortalitet og myokardie(re)infarkt hos patienter med ustabil angina eller non-Q-tak-myokardieinfarkt.

ESPRIT: Dette var en dobbeltblindet, multicenter, randomiseret, parallelgruppe, placebokontrolleret undersøgelse, hvor virkning og sikkerhed af behandling med eptifibatid blev vurderet hos patienter, hvor perkutan koronar-intervention (PCI) med intrakoronar stent var planlagt.

I PURSUIT blev blødningshændelser og ikke-blødningshændelser samlet fra hospitalsudskrivelse og indtil kontrol 30 dage efter. I ESPRIT blev blødningshændelser rapporteret ved 48 timer og ikke-blødningshændelser ved 30 dage. Mens Thrombolysis in Myocardial Infarction (TIMI) study group kriterierne blev anvendt til klassifikation af incidensen af større og mindre blødninger i både PURSUIT- og ESPRIT-undersøgelsen, blev PURSUIT-data indsamlet indenfor 30 dage, hvor ESPRIT-data var begrænset til hændelser indenfor 48 timer eller ved udskrivning, afhængig af hvad der skete først.

Bivirkningerne er inddelt efter systemorganklasse og hyppighed. Hyppigheden er defineret som: Meget almindelig (≥1/10), Almindelig (≥1/100 til <1/10), Ikke almindelig (≥1/1000 til <1/100), Sjælden (≥1/10.000 til <1/1000), Meget sjælden (<1/10.000), Ikke kendt (kan ikke estimeres ud fra tilgængelige data). Disse er absolutte bivirkningsfrekvenser uden hensyntagen til placebofrekvens. Hvis en specifik bivirkning blev rapporteret i både PURSUIT og ESPRIT, blev den højest rapporterede hændelse anvendt til bestemmelse af bivirkningsfrekvensen.

Bemærk at årsagssammenhæng ikke er fastlagt for alle bivirkninger.

Blod og lymfesystem
Meget almindelig: Blødninger (større og mindre blødninger inklusive femoral arterieindstikssted, CABG-relateret, gastrointestinal, genital/urinal, retroperitoneal, intrakraniel, hæmatemese, hæmaturi, oral/orofaryngeal, hæmoglobin-/hæmatokritreduktion og andre).

Ikke almindelig: Thrombocytopeni.

Nervesystemet
Ikke almindelig: Cerebral iskæmi.

Hjerte
Almindelig: Hjertestop, ventrikelflimren, ventrikulær takykardi, hjerteinsufficiens, atrioventrikulært blok, atrieflimren.

Vaskulære sygdomme:
Almindelig: Shock, hypotension, phlebitis.

Hjertestop, hjerteinsufficiens, atrieflimren, hypotension og shock, som er almindeligt rapporterede hændelser fra PURSUIT undersøgelsen, var hændelser relateret til den bagvedliggende sygdom.

Administration af eptifibatid er forbundet med en stigning i antallet af større og mindre blødninger, klassificeret efter TIMI study group kriterierne. Ved den anbefalede terapeutiske dosis, som blev anvendt i PURSUIT-undersøgelsen med næsten 11.000 patienter, var blødning den hyppigste komplikation, der forekom under

behandling med epfibatid. De hyppigste blødningskomplikationer var forbundet med invasive hjerteprocedurer (koronararterie bypass-kirurgi (CABG)-relaterede eller ved femoral arterieindstiksssted).

Mindre blødninger blev i PURSUIT-undersøgelsen defineret som spontan kraftig hæmaturi, spontan hæmatemese, observeret blodtab med et fald i hæmoglobin på mere end 3 g/dl eller mere end 4 g/dl uden observeret blødningssted. Under behandling med Integrilin i denne undersøgelse var mindre blødning en meget almindelig komplikation (> 1/10 eller 13,1 % for Integrilin mod 7,6 % for placebo). Blødningshændelser var hyppigere hos patienter, der samtidig fik heparin under gennemførelse af PCI, når ACT oversteg 350 sekunder (se pkt. 4.4, heparin-indgift).

Større blødninger blev i PURSUIT-undersøgelsen defineret som enten en intrakraniel blødning eller et fald i hæmoglobin-koncentrationer på mere end 5 g/dl. Større blødninger var også en meget almindelig komplikation og blev rapporteret hyppigere hos de patienter der blev behandlet med Integrilin end hos dem, der fik placebo i PURSUIT-undersøgelsen (> 1/10 eller 10,8 % mod 9,3 %), men det var ikke almindeligt hos størstedelen af de patienter, der ikke gennemgik CABG indenfor 30 dage efter inklusion i studiet. Hos patienter, der gennemgik CABG, øgede Integrilin ikke forekomsten af blødninger sammenlignet med patienter behandlet med placebo. I undergruppen med patienter, der gennemgik PCI var observation af større blødninger almindelig hos 9,7 % af de patienter, der blev behandlet med eptifibatid, sammenlignet med 4,7 % af de placebobehandlede patienter.

Forekomsten af svære eller livstruende blødningshændelser med Integrilin var 1,9 % sammenlignet med 1,1 % med placebo. Behandling med Integrilin øgede i moderat grad behovet for blodtransfusioner (11,8 % mod 9,3 % for placebo).

Ændringer under behandling med eptifibatid er følger af den kendte farmakologiske virkning, det vil sige hæmning af trombocytaggregationen. Ændringer i de laboratorieparametre, der associeres med blødninger (som blødningstiden) er således almindelige og forventelige. Hvad angår værdierne for leverfunktionen (SGOT/AST, SGPT/ALAT, bilirubin, alkalisk fosfatase) og værdierne for nyrefunktionen (serumkreatinin, blod-urea-nitrogen), observeredes der ingen tilsyneladende forskelle på de patienter, der blev behandlet med eptifibatid og dem, der fik placebo.

Erfaringer efter markedsføring

Blod og lymfesystem
Meget sjælden: fatal blødning (størstedelen involverede sygdomme i det centrale og perifere nervesystem: cerebrale eller intrakranielle blødninger); pulmonale blødninger; akut kraftig trombocytopeni, hæmatom, anæmi.

Immunsystemet
Meget sjælden: anafylaktiske reaktioner.

Hud og subkutane væv
Meget sjælden: udslæt, reaktioner på indgivelsesstedet såsom urticaria.

4.9 Overdosering

Erfaring med overdosering af eptifibatid hos mennesker er yderst begrænset. Der var ingen tegn på svære bivirkninger forbundet med tilfældig indgift af store bolusdoser, hurtig infusion rapporteret som overdosering eller store kumulative doser. I PURSUIT-undersøgelsen var der 9 patienter, som fik bolus- og/eller infusionsdoser, der var mere end dobbelt så store som den anbefalede dosis, eller som blev identificeret af investigator til at have modtaget en overdosering. Der var ingen øget blødning hos nogen af disse patienter, selv om der er indberettet et tilfælde af moderat blødning under en CABG-operation. Helt specifikt fik ingen patienter en hjerneblødning.

En overdosering af eptifibatid kan potentielt resultere i blødninger. På grund af dets korte halveringstid og hurtige clearance kan virkningen af eptifibatid hurtigt stoppes ved at afbryde infusionen. Selv om eptifibatid kan dialyseres, er det usandsynligt, at der skulle opstå behov for dialyse.

5. FARMAKOLOGISKE EGENSKABER

5.1 Farmakodynamiske egenskaber

Farmakoterapeutisk klassifikation: Antitrombotisk middel (trombocytaggregationshæmmere, eksklusive heparin), ATC-kode: B01AC16

Eptifibatid, et syntetisk cyklisk heptapeptid indeholdende seks aminosyrer, som omfatter et cysteinamid og en merkaptopropionyl-rest (desaminocysteinyl-), er en trombocytaggregationshæmmer i klassen RGD(arginin-glycin-aspartat)-mimetika.

Eptifibatid hæmmer trombocytaggregationen reversibelt ved at forebygge bindingen af fibrinogen, von-Willebrand faktor og andre adhæsive ligander til glykoprotein (GP) IIb/IIIa-receptorer.

Eptifibatid hæmmer trombocytaggregationen på en dosis- og koncentrationsafhængig måde, som demonstreret ved *ex vivo* trombocytaggregation ved anvendelse af adenosindiphosphat (ADP) og andre agonister for induktion af pladeaggregation. Virkningen af eptifibatid ses øjeblikkeligt efter indgift af en 180 mikrogram/kg intravenøs bolus. Ved efterfølgende 2,0 mikrogram/kg/minut kontinuerlig infusion giver dette regimen en > 80 % hæmning af ADP-induceret *ex vivo* trombocytaggregation, ved fysiologiske calciumkoncentrationer, hos mere end 80 % af patienterne.

Trombocythæmningen var hurtig reversibel med en genetablering af blodpladefunktionen mod udgangsværdien (> 50 % pladeaggregation) 4 timer efter afbrydelse af en kontinuerlig infusion på 2,0 mikrogram/kg/minut. Målinger af ADP-induceret *ex vivo* trombocytaggregation ved fysiologiske calciumkoncentrationer (D-phenylalanyl-L-prolyl-L-arginin chloromethyl keton antikoagulant) hos patienter med ustabil angina og non-Q-tak-myokardieinfarkt viste en koncentrationsafhængig hæmning med en IC_{50} (50 % hæmmende koncentration) på omkring 550 ng/ml og en IC_{80} (80 % hæmmende koncentration) på omkring 1.100 ng/ml.

PURSUIT-undersøgelsen
Den grundlæggende kliniske undersøgelse ved ustabil angina (UA)/non-Q-tak-myokardieinfarkt (NQMI) var PURSUIT. Denne undersøgelse var en 726-centre, 27-lande, dobbeltblind, randomiseret, placebokontrolleret undersøgelse af 10.948 patienter, der havde UA eller NQMI. Patienterne kunne kun inkluderes, hvis de havde haft hjerteiskæmi i hvile (≥ 10 minutter) inden for de sidste 24 timer og havde:
enten ST-segmentændringer: ST-depression > 0,5 mm i mindre end 30 minutter eller vedvarende ST-forhøjelse > 0,5 mm, der ikke krævede reperfusionsbehandling eller trombolytiske midler, T-takinversion (> 1 mm)
eller forhøjet CK-MB.

Patienterne blev randomiseret til enten placebo, eptifibatid180 mikrogram/kg bolus efterfulgt af en 2,0 mikrogram/kg/minut infusion (180/2,0) eller eptifibatid 180 mikrogram/kg bolus efterfulgt af en 1,3 mikrogram/kg/minut infusion (180/1,3).

Infusionen blev fortsat indtil udskrivning fra hospitalet, indtil tidspunkt for koronararterie bypass (CABG) eller i op til 72 timer, efter hvad der forekom først. Ved PCI blev infusionen med eptifibatid fortsat i 24 timer efter proceduren med en infusionsvarighed op til 96 timer.

Gruppen 180/1,3 blev stoppet efter en interimanalyse, som tidligere anført i protokollen, da de to aktive behandlingsarme syntes at have samme blødningsfrekvens.

Patienterne blev behandlet ifølge de sædvanlige standarder på undersøgelsesstedet. Hyppigheden af angiografi, PCI og CABG var derfor meget forskellig fra sted til sted og fra land til land. 13 % af patienterne i PURSUIT blev behandlet med PCI under infusionen med eptifibatid, heraf fik cirka 50 % intrakoronare stents, og 87 % blev behandlet medicinsk (uden PCI under infusionen med eptifibatid).

De fleste patienter fik acetylsalicylsyre (75-325 mg en gang daglig).
Ufraktioneret heparin blev indgivet intravenøst eller subkutant efter lægens skøn, oftest som en intravenøs bolus på 5.000 enheder efterfulgt af en kontinuerlig infusion af 1.000 enheder/time. Et mål for aPTT på 50-70 sekunder blev anbefalet. I alt 1.250 patienter gennemgik PCI inden for 72 timer efter randomisering, og de modtog da intravenøs ufraktioneret heparin for at vedligeholde en aktiveret koagulationstid (ACT) på 300-350 sekunder.

Det primære endpoint for undersøgelsen var forekomst af dødsfald uafhængigt af årsagen eller nyt myokardieinfarkt (MI) (vurderet af en blindet data monitorerings komité) inden for 30 dage efter randomisering. Komponenten MI kunne defineres som asymptomatisk med enzymatisk stigning i CK-MB eller ny Q-tak.

Sammenlignet med placebo reducerede eptifibatid indgivet som 180/2,0 signifikant hyppigheden af primære endpoint-hændelser (Tabel 1); dette svarer til 15 undgåede hændelser per 1000 patienter behandlet:

Tabel 1.
Hyppigheden af dødsfald/CEC-vurderede MI ("Behandlet som randomiseret" population)

Tid	Placebo	Eptifibatid	p-værdi
30 dage	743/4.697 (15,8 %)	667/4.680 (14,3 %)	0,034[a]

[a]: Pearson's chi-square test af difference mellem placebo og eptifibatid.

Resultaterne af det primære endpoint blev hovedsageligt tillagt fremkomsten af myokardieinfarkt. Reduktionen i hyppigheden af endpoint-hændelser hos patienter, som fik eptifibatid, optrådte tidligt i behandlingsforløbet (inden for de første 72-96 timer). Denne reduktion blev vedligeholdt i 6 måneder uden nogen signifikant virkning på mortaliteten.

De patienter, som med størst sandsynlighed vil have gavn af behandling med eptifibatid, er dem med høj risiko for at udvikle myokardieinfarkt inden for de første 3-4 dage efter debut af den akutte angina.
Ifølge epidemiologiske fund er en højere hyppighed af kardiovaskulære hændelser knyttet til visse indikatorer, fx:
alder
øget puls eller blodtryk
vedvarende eller tilbagevendende iskæmiske hjertesmerter
udtalte ekg-forandringer (især ST-segmentanomalier)
forhøjede hjerteenzymer eller markører (fx CK-MB, troponiner) og
hjerteinsufficiens

ESPRIT-undersøgelsen
ESPRIT (Enhanced Suppression of the Platelet IIb/IIIa Receptor with Eptifibatid Therapy: Forstærket supprimering af blodpladereceptor IIb/IIIa med behandling med eptifibatid) var en dobbeltblind, randomiseret, placebokontrolleret undersøgelse (n=2.064) af ikke akut PCI med intrakoronar stent.

Alle patienter fik standardbehandling og blev randomiseret til enten placebo eller eptifibatid (2 bolus- doser på 180 mikrogram/kg og en kontinuerlig infusion indtil udskrivelse fra hospitalet eller højst 18-24 timer).

Den første bolus og infusionen blev startet samtidigt, umiddelbart før PCI-proceduren og blev efterfulgt af en yderligere bolus 10 minutter efter den første. Infusionshastigheden var 2,0 mikrogram/kg/minut til patienter med serumkreatinin ≤ 175 mikromol/l eller 1,0 mikrogram/kg/minut ved serumkreatinin > 175 op til 350 mikromol/l.

I armen med eptifibatid i undersøgelsen fik næsten alle patienter acetylsalicylsyre (99,7 %), og 98,1 % fik thienopyridin (clopidogrel hos 95,4 % og ticlopidin hos 2,7 %). På dagen for PCI, før kateterindføringen, fik 53,2 % et thienopyridin (clopidogrel 52,7 %; ticlopidin 0,5 %) – oftest som en høj initialdosis (300 mg eller mere). Placeboarmen var tilsvarende (acetylsalicylsyre 99,7 %, clopidogrel 95,9 %, ticlopidin 2,6 %).

ESPRIT-undersøgelsen anvendte et simplificeret heparin-regimen under PCI, som bestod af en initial bolus på 60 enheder/kg, med en målsætning for ACT på 200-300 sekunder. Undersøgelsens primære effektmål var død (D), MI, akut revaskularisering af arterien (UTVR), og akut antitrombotisk reddende behandling med GP IIb/IIIa inhibitor (RT) inden for 48 timer efter randomiseringen.

MI blev påvist ved CK-MB-laboratorieundersøgelseskriterier. For denne diagnose skulle der inden for 24 timer efter den registrerede PCI-procedure være mindst to CK-MB-værdier ≥ 3 x den øvre grænse af normalområdet. Bekræftelse fra den blindede sikkerhedskomité var således ikke påkrævet. MI kunne også rapporteres efter bedømmelse fra den blindede sikkerhedskomité af en investigator- rapport.

Den primære effektmålsanalyse [firesidet sammensætning af død, MI, akut revaskularisering af arterien (UTVR) og trombolytisk redningsaktion (TBO) efter 48 timer] viste 37 % relativ og 3,9 % absolut reduktion i eptifibatid-gruppen (6,6 % hændelser imod 10,5 %, p = 0,0015). Resultaterne af det primære effektmål var hovedsageligt kendetegnet ved reduktion i forekomsten af enzymatisk MI, identificeret som forekomsten af tidlig forøgelse af kardielle enzymer efter PCI (80 ud af 92 myokardieinfarkter i placebogruppen over for 47 ud af 56 i eptifibitid-gruppen). Den kliniske relevans af sådanne enzymatiske myokardieinfarkter er stadig kontroversiel.

Lignende resultater blev også opnået for de 2 sekundære effektmål vurderet efter 30 dage: en tresidet sammensætning af død, MI og UTVR, og den stærkere kombination af død og MI.

Reduktionen i forekomsten af effektmålshændelser hos patienter, der modtog eptifibatid, viste sig tidligt i behandlingen. Der var ikke yderligere effekt derefter i op til 1 år.

Forlænget blødningstid
Indgift af eptifibatid ved intravenøs bolus og infusion forårsager op til 5 gange stigning i blødningstiden. Denne stigning er hurtig reversibel efter seponering af infusionen, og blødningstiden vender tilbage til udgangsværdien inden for cirka 6 (2-8) timer. Eptifibatid, indgivet alene, har ingen målelig effekt på protrombintiden (PT) eller aktiveret partiel tromboplastintid (aPTT).

5.2 Farmakokinetiske egenskaber

Eptifibatids farmakokinetik er lineær og dosisproportional for bolusdoser i området fra 90 til 250 mikrogram/kg og infusionshastigheder fra 0,5 til 3,0 mikrogram/kg/minut. Ved en 2,0 mikrogram/kg/minut infusion opnås gennemsnitlige steady-state eptifibatid-plasmakoncentrationer fra 1,5 til 2,2 mikrogram/ml hos patienter med koronararteriesygdom. De nævnte plasmakoncentrationer opnås hurtigt, når infusionen forudgås af en 180 mikrogram/kg bolusinjektion. Graden af eptifibatid binding til humant plasmaprotein er omkring 25 %. I samme population er plasmaeliminationshalveringstiden cirka 2,5 timer, plasmaclearance 55 til 80 ml/kg/time og -+fordelingsvolumen cirka 185 til 260 ml/kg.

Hos raske personer udgjorde den renale udskillelse cirka 50 % af den totale udskillelse fra kroppen, cirka 50 % af den udskilte mængde udskilles uomdannet. Hos patienter med moderat til svær nyreinsufficiens (kreatininclearance < 50 ml/minut) er clearance reduceret med cirka 50 % og steady-state plasmaværdier omtrent fordoblet. Der er ikke blevet udført nogen egentlige farmakokinetiske interaktionsundersøgelser. I en populationsfarmakokinetisk undersøgelse var der imidlertid ingen tegn på farmakokinetisk interaktion mellem eptifibatid og følgende samtidigt indgivne præparater: amlodipin, atenolol, atropin, captopril, cefazolin, diazepam, digoxin, diltiazem, diphenhydramin, enalapril, fentanyl, furosemid, heparin, lidocain, lisinopril, metoprolol, midazolam, morphin, nitrater, nifedipin og warfarin.

5.3 Prækliniske sikkerhedsdata

Toksikologiundersøgelser udført med eptifibatid omfatter undersøgelser med gentagne doser hos rotter, kaniner og aber, reproduktionsundersøgelser hos rotter og kaniner, *in vitro* og *in vivo* genetiske toksicitetsundersøgelser og irritations-, overfølsomheds- og antigenicitetsundersøgelser. Ingen uventede toksiske virkninger for et stof med denne farmakologiske profil blev observeret, og fundene var prediktive for klinisk erfaring med blødningshændelser som værende den hovedsagelige bivirkning. Ingen genotoksiske virkninger blev observeret med eptifibatid.

Teratologiske undersøgelser er udført med kontinuerlig intravenøs infusion af eptifibatid til drægtige rotter med totale daglige doser på op til 72 mg/kg/dag (omkring 4 gange den anbefalede maksimale humane dosis baseret på legemsoverfladeareal), og til drægtige kaniner med totale daglige doser på op til 36 mg/kg/dag (omkring 4 gange den anbefalede maksimale humane dosis baseret på legemsoverfladeareal). Disse undersøgelser viste ingen tegn på forringet fertilitet eller fosterskade på grund af eptifibatid. Reproduktionsundersøgelser på dyr, hvor eptifibatid viser en lignende farmakologisk aktivitet som hos mennesker, findes ikke. Ovennævnte undersøgelser er således ikke velegnede til at vurdere toksiciteten af eptifibatid på reproduktionsfunktionen (se pkt 4.6).

Det karcinogene potentiale for eptifibatid er ikke blevet vurderet i langtidsundersøgelser.

6. FARMACEUTISKE OPLYSNINGER

6.1 Hjælpestoffer

Citronsyremonohydrat
Natriumhydroxid
Vand til injektionsvæsker

6.2 Uforligeligheder

INTEGRILIN er ikke forligeligt med furosemid.
Da der ikke foreligger undersøgelser vedrørende eventuelle uforligeligheder, bør INTEGRILIN ikke blandes med andre lægemidler end dem, der er anført under pkt. 6.6.

6.3 Opbevaringstid

3 år

6.4 Særlige opbevaringsforhold

Opbevares i køleskab (2 °C - 8 °C). Opbevares i den originale yderpakning for at beskytte mod lys.

6.5 Emballagetype og pakningsstørrelser

Et 10 ml Type I glas hætteglas, lukket med en butylgummiprop og forseglet med et krympet aluminumsegl.

6.6 Regler for destruktion og anden håndtering

Fysiske og kemiske forligelighedsforsøg indicerer, at INTEGRILIN kan indgives via en intravenøs linje med atropinsulfat, dobutamin, heparin, lidocain, meperidin, metoprolol, midazolam, morphin, nitroglycerin, vævs plasminogen aktivator eller verapamil. INTEGRILIN er forligeligt med 0,9 % natriumchlorid injektionsvæske og med glucose 5 % i Normosol R med eller kaliumchlorid. Se produktresumé for Normosol R for detaljer om sammensætning.

Før anvendelsen inspiceres indholdet i hætteglasset. Anvend ikke præparatet ved tegn på bundfald eller misfarvning. Det er ikke nødvendigt at beskytte INTEGRILIN-opløsning mod lys under selve indgivelsen.

Ikke anvendt lægemiddel skal bortkastes efter åbning.

7. INDEHAVER AF MARKEDSFØRINGSTILLADELSEN

Glaxo Group Ltd
Greenford
Middlesex
UB6 0NN
Storbritannien

8. MARKEDSFØRINGSTILLADELSESNUMMER (NUMRE)

EU/1/99/109/002

9. DATO FOR FØRSTE MARKEDSFØRINGSTILLADELSE/FORNYELSE AF TILLADELSEN

Dato for første MT: 01.07.1999
Dato for fornyelse: 09.07.2009

10. DATO FOR ÆNDRING AF TEKSTEN

Yderligere information om dette lægemiddel er tilgængelig på Det europæiske Lægemiddelagenturs (EMEA's) hjemmeside http://www.emea.europa.eu/
