# Supplementary material for: Co-administration of iloprost and eptifibatide in septic shock (CO-ILEPSS)—a randomised, controlled, double-blind investigator-initiated trial investigating safety and efficacy
Source: Crit Care. 2019 Sep 5;23:301. doi: 10.1186/s13054-019-2573-8 (PMC6727583; doi:10.1186/s13054-019-2573-8)
Supplement: Supplementary file 3 — In- and exclusion criteria. (PDF 369 kb) [file 13054_2019_2573_MOESM3_ESM.pdf]

Additional file 3 – In- and exclusion criteria

Inclusion criteria

1. Adult intensive care patients (age ≥18 years)
   AND
2. Sepsis, defined as suspected or confirmed site of infection or positive blood culture and ≥2 of 4 systemic inflammatory response syndrome (SIRS) criteria fulfilled within the last 24h:
   a. Temperature ≤ 36° C or ≥ 38°C
   b. Heart rate ≥ 90 beats per minute
   c. Mechanical ventilation for acute respiratory process or respiratory rate ≥ 20 breaths per minute or PaCO2 < 4.2 kPa
   d. WBC ≥ 12,000/mm³ OR ≤ 4,000/mm³ OR > 10% bands

   AND
3. Septic shock within the last 24h, defined as:
   a. Hypotension (MAP <70 mmHg, Lactate 4 mmol/L) despite ongoing resuscitation with fluids (crystalloids, colloids, blood products) within the last 24h OR
      OR
   b. ≥30 ml/kg ideal body weight (IBW) fluid (crystalloids, colloids, blood products) given in the last 24h
      AND
   c. Need for vasopressor/inotropic agents (noradrenaline, adrenaline, dopamine) within the last 24h

   AND
4. Can be randomized into trial and dosed < 24h after septic shock diagnosis (the time-point for the septic shock diagnosis corresponds to the time-point where the vasopressor/inotropic therapy (3c) is initiated)
   AND
5. Consent is obtainable

Exclusion criteria

Patients are not eligible for inclusion in this trial if they fulfill one or more of the following criteria:

1. Pregnant or breast-feeding
2. Weight more than 125 kg
3. Known allergy towards any of the investigational products or contraindications which should be excluded according to the investigational product specifications
4. Patient in whom the clinician finds antithrombotic therapy contraindicated - prophylaxis included
5. Increased risk of bleeding:
   a. Surgery in the previous 48h and expected surgery within 48 h
   b. Epidural or spinal puncture in the previous 12h
   c. Platelet count less than 10,000/mm3 in the previous 24h
   d. Need of blood products for bleeding in the previous 24h (3 or more RBC/24 h)
   e. Treatment with any antithrombotics within 12h (profylaxis excepted)
   f. Current intracranial bleeding
   g. Traumatic brain or spinal injury within the last month
6. Treatment with any form of antithrombotics (beyond profylaxis) in therapeutic doses or prothrombotics in any dose, including:

    a. Unfractionated heparin within 8h before the infusion (prophylactic heparin up to 15,000 U/day permitted)
    b. LMWH within 12h before the infusion (prophylactic doses permitted)
    c. Warfarin within 1 day before the infusion
    d. Acetylsalicylic acid more than 650 mg/day within 3 days before the study
    e. Thrombolytic therapy within 3 days before the study (catheter clearance doses permitted)
    f. GPIIb/IIIa receptor inhibitors within 4 days before the study
    g. Antithrombin III with dose greater than 10,000 U within 12h before the study
7. Do-not-resuscitate order (expected not to survive more than few days because of uncorrectable medical or surgical condition other than sepsis)
8. Chronic renal failure requiring dialysis (renal failure without need for dialysis permitted)
9. Patient who have undergone transplantation of bone marrow, liver, pancreas, heart, lung or bowel (kidney transplant permitted)
10. Known hypercoagulable condition:
    a. Activated protein C resistance
    b. Hereditary protein C, protein S, or antithrombin III deficiency
    c. Anticardiolipin or antiphospholipid antibody
    d. Lupus anticoagulant
    e. Homocysteinemia
    f. Recent or highly suspected pulmonary embolism or deep venous thrombosis (within 3 months)
11. Known congenital hypocoagulable diseases
12. Known primary pulmonary hypertension
