# Supplementary figures and images for: Co-administration of iloprost and eptifibatide in septic shock (CO-ILEPSS)—a randomised, controlled, double-blind investigator-initiated trial investigating safety and efficacy
Source: Crit Care. 2019 Sep 5;23:301. doi: 10.1186/s13054-019-2573-8 (PMC6727583; doi:10.1186/s13054-019-2573-8)

Additional file 4 – Relative biomarker measurements

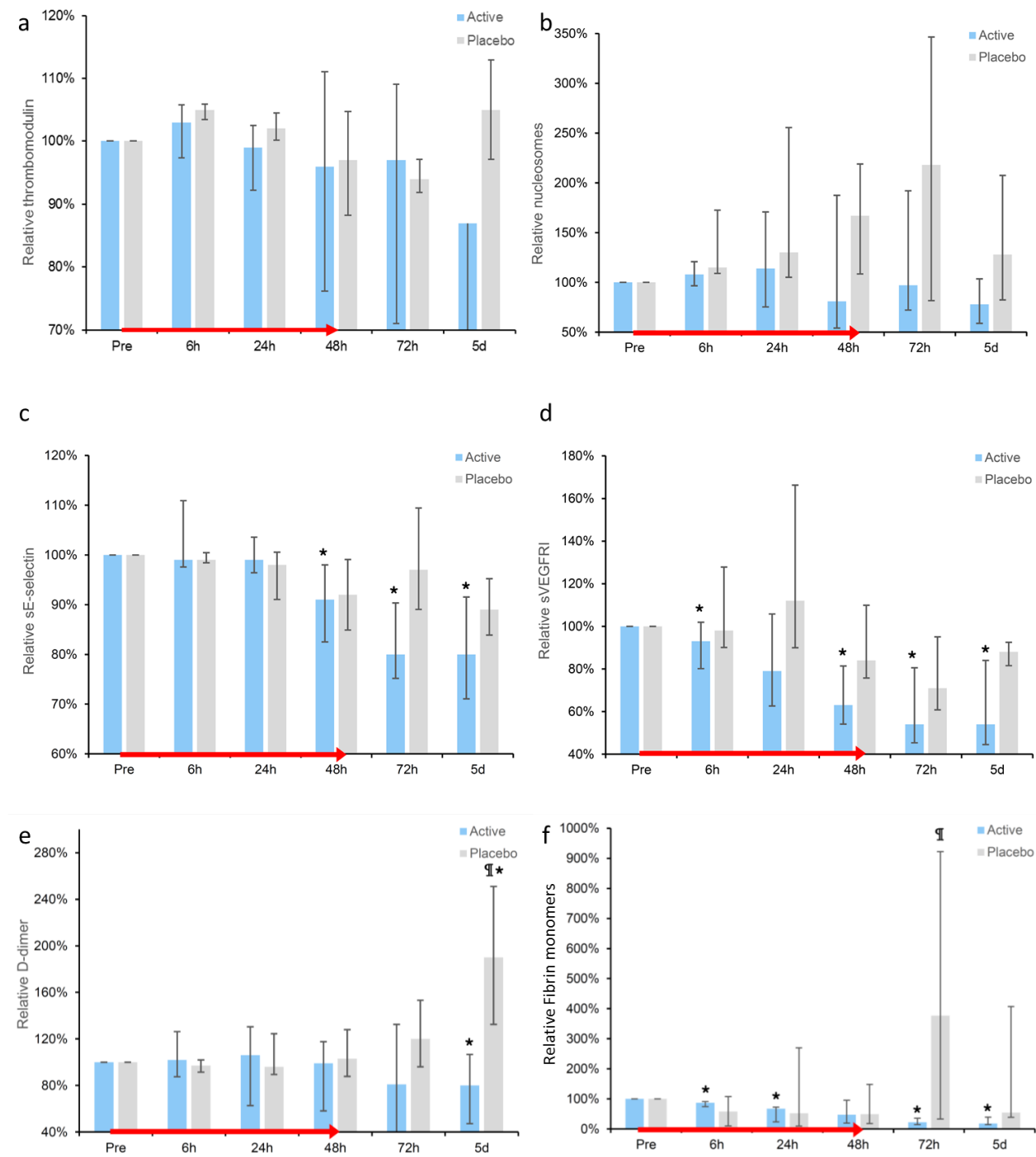

Supplement: Supplementary file 4 — Relative biomarker measures. (PDF 540 kb) [file 13054_2019_2573_MOESM4_ESM.pdf]
