# Supplementary material for: Co-administration of iloprost and eptifibatide in septic shock (CO-ILEPSS)—a randomised, controlled, double-blind investigator-initiated trial investigating safety and efficacy
Source: Crit Care. 2019 Sep 5;23:301. doi: 10.1186/s13054-019-2573-8 (PMC6727583; doi:10.1186/s13054-019-2573-8)
Supplement: Supplementary file 5 — Secondary endpoints. (PDF 640 kb) [file 13054_2019_2573_MOESM5_ESM.pdf]

Additional file 5 – Secondary outcomes

Mortality

Secondary endpoint

Intention to treat (n=24)	Active drug (n=15)	Placebo (n=9)	p-value
7-day	0 (0%)	2 (22%)	0.130
30-day	2 (13%)	4 (44%)	0.150
90-day	4 (27%)	5 (56%)	0.212

Per-protocol (n=18)	Active drug (n=12)	Placebo (n=6)	p-value
7-day	0 (0%)	1 (17%)	0.333
30-day	1 (8%)	2 (32%)	0.254
90-day	3 (25%)	3 (50%)	0.294

Tendency towards lover mortality in the active treatment group in the *Intention-to-Treat* group. No difference in mortality between the active treatment and placebo groups in the *Per-Protocol* group.

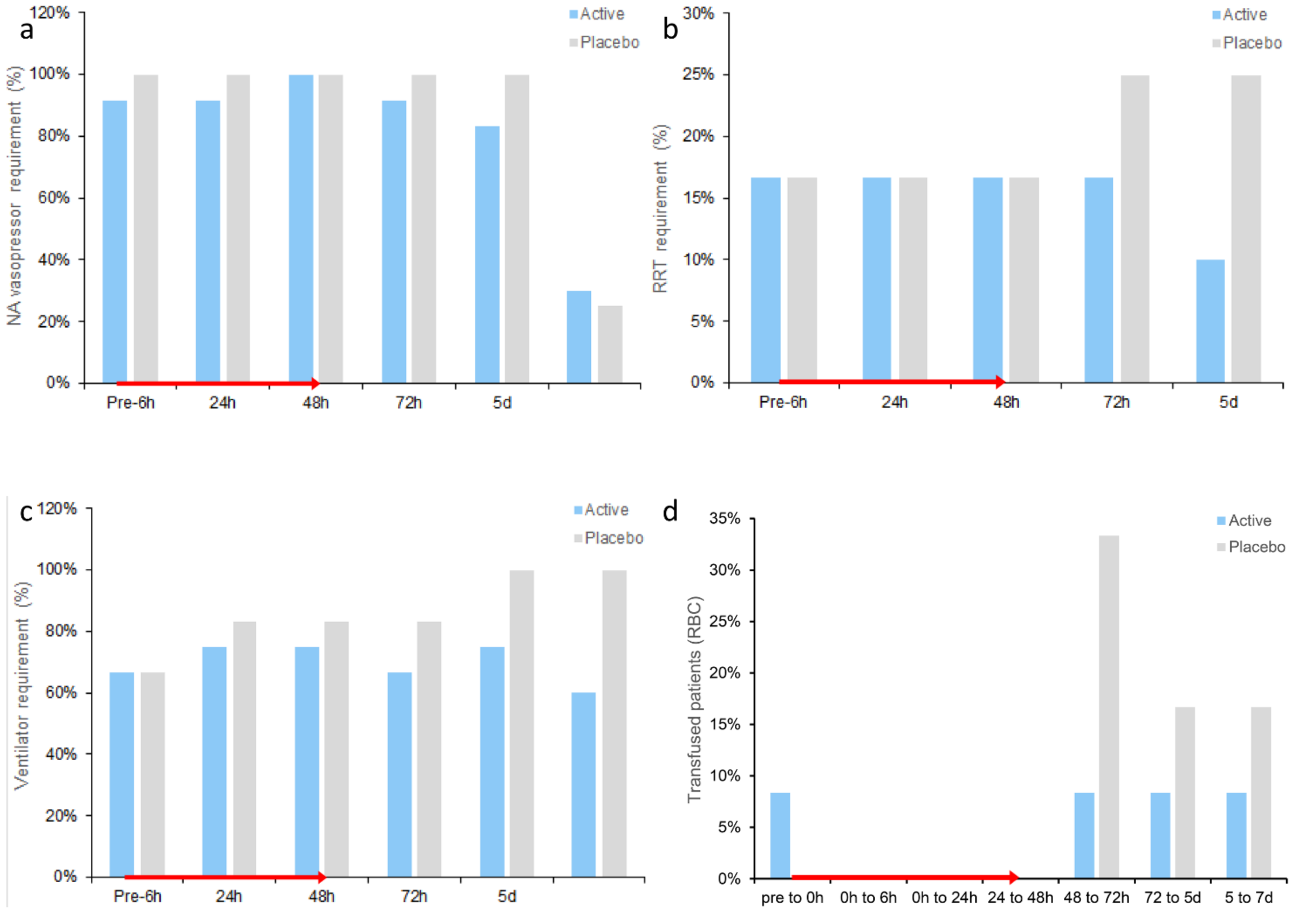

Data shown as median proportion (%) of patients.

NA = Noradrenaline, RRT = Renal replacement therapy, RBC = Red Blood Cells.
